# Normative spatiotemporal fetal brain maturation with satisfactory development at 2 years

**DOI:** 10.1038/s41586-023-06630-3

**Published:** 2023-10-25

**Authors:** Ana I. L. Namburete, Bartłomiej W. Papież, Michelle Fernandes, Madeleine K. Wyburd, Linde S. Hesse, Felipe A. Moser, Leila Cheikh Ismail, Robert B. Gunier, Waney Squier, Eric O. Ohuma, Maria Carvalho, Yasmin Jaffer, Michael Gravett, Qingqing Wu, Ann Lambert, Adele Winsey, María C. Restrepo-Méndez, Enrico Bertino, Manorama Purwar, Fernando C. Barros, Alan Stein, J. Alison Noble, Zoltán Molnár, Mark Jenkinson, Zulfiqar A. Bhutta, Aris T. Papageorghiou, José Villar, Stephen H. Kennedy

**Affiliations:** 1 Oxford Machine Learning in Neuroimaging Laboratory, Department of Computer Science, University of Oxford, Oxford, UK; 2 Wellcome Centre for Integrative Neuroimaging, University of Oxford, Oxford, UK; 3 Department of Engineering Science, University of Oxford, Oxford, UK; 4 Big Data Institute, Li Ka Shing Centre for Health Information and Discovery, University of Oxford, Oxford, UK; 5 Nuffield Department of Women’s and Reproductive Health, University of Oxford, Oxford, UK; 6 MRC Lifecourse Epidemiology Centre, Human Development and Health Academic Unit, Faculty of Medicine, University of Southampton, Southampton, UK; 7 Oxford Maternal and Perinatal Health Institute, Green Templeton College, University of Oxford, Oxford, UK; 8 Department of Clinical Nutrition and Dietetics, College of Health Sciences, University of Sharjah, Sharjah, United Arab Emirates; 9 Center for Environmental Research and Children’s Health, School of Public Health, University of California, Berkeley, CA USA; 10 Department of Neuropathology, John Radcliffe Hospital, Oxford, UK; 11 Maternal, Adolescent, Reproductive and Child Health Centre, London School of Hygiene and Tropical Medicine, London, UK; 12 Department of Obstetrics and Gynaecology, Faculty of Health Sciences, Aga Khan University Hospital, Nairobi, Kenya; 13 Department of Family and Community Health, Ministry of Health, Muscat, Sultanate of Oman; 14 Departments of Obstetrics and Gynecology and of Global Health, University of Washington, Seattle, WA USA; 15 School of Public Health, Peking University, Beijing, China; 16 Dipartimento di Scienze Pediatriche e dell’ Adolescenza, SCDU Neonatologia, Universita di Torino, Turin, Italy; 17 Nagpur INTERGROWTH-21st Research Centre, Ketkar Hospital, Nagpur, India; 18 Programa de Pós-Graduação em Saúde e Comportamento, Universidade Católica de Pelotas, Pelotas, Brazil; 19 Department of Psychiatry, University of Oxford, Oxford, UK; 20 African Health Research Institute, KwaZulu-Natal, South Africa; 21 MRC/Wits Rural Public Health and Health Transitions Research Unit (Agincourt), School of Public Health, Faculty of Health Sciences, University of Witwatersrand, Johannesburg, South Africa; 22 Department of Physiology, Anatomy and Genetics, University of Oxford, Oxford, UK; 23 Australian Institute for Machine Learning, Department of Computer Science, University of Adelaide, Adelaide, South Australia Australia; 24 South Australian Health and Medical Research Institute, Adelaide, South Australia Australia; 25 Center for Global Child Health, Hospital for Sick Children, Toronto, Ontario Canada

**Keywords:** Intrauterine growth, Medical imaging, Neuroscience

## Abstract

Maturation of the human fetal brain should follow precisely scheduled structural growth and folding of the cerebral cortex for optimal postnatal function^1^. We present a normative digital atlas of fetal brain maturation based on a prospective international cohort of healthy pregnant women^2^, selected using World Health Organization recommendations for growth standards^3^. Their fetuses were accurately dated in the first trimester, with satisfactory growth and neurodevelopment from early pregnancy to 2 years of age^4,5^. The atlas was produced using 1,059 optimal quality, three-dimensional ultrasound brain volumes from 899 of the fetuses and an automated analysis pipeline^6–8^. The atlas corresponds structurally to published magnetic resonance images^9^, but with finer anatomical details in deep grey matter. The between-study site variability represented less than 8.0% of the total variance of all brain measures, supporting pooling data from the eight study sites to produce patterns of normative maturation. We have thereby generated an average representation of each cerebral hemisphere between 14 and 31 weeks’ gestation with quantification of intracranial volume variability and growth patterns. Emergent asymmetries were detectable from as early as 14 weeks, with peak asymmetries in regions associated with language development and functional lateralization between 20 and 26 weeks’ gestation. These patterns were validated in 1,487 three-dimensional brain volumes from 1,295 different fetuses in the same cohort. We provide a unique spatiotemporal benchmark of fetal brain maturation from a large cohort with normative postnatal growth and neurodevelopment.

## Abstract (web summary)

A normative digital atlas of fetal brain maturation produced using 1,059 optimal quality, three-dimensional ultrasound brain volumes from 899 fetuses presents a unique spatiotemporal benchmark from a large cohort with normative postnatal growth and neurodevelopment at 2 years of age.

## Main

Constructing a spatial map of the human fetal brain, which is essential for relating structural organization to functionality, was first achieved with cross-sectional data from postmortem observations^10^, obviously without any postnatal follow-up. The data described a process of cortical folding and volumetric growth that follows a spatiotemporal schedule, commencing in the second trimester of pregnancy. Thereafter, the cortical surface rapidly increases in complexity as sulcal folds and gyri emerge with brain morphology continuing to change after birth^11^.

These structural changes require an intricate sequence of cellular proliferation and neuronal migration^12^, underpinning the functional specialization seen in later life. Severe disruption of the processes in early pregnancy causes major congenital malformations and loss of function, as described, for example, due to infection with the Zika virus^13^, but the degree of normal variability in fetal brain maturation, associated with adequate postnatal growth and neurodevelopment, is uncertain.

Several structural fetal magnetic resonance imaging (MRI) studies have confirmed postmortem findings, and revealed spatiotemporal patterns of development^1,14–17^, asymmetry^16,18–20^, sex differences^16,21^ and, more recently, volumetric growth^20,22^. However, these studies are limited by their single laboratory design, small sample sizes (range 12–197), methodological heterogeneity and lack of postnatal follow-up into childhood. Furthermore, the strict prescriptive criteria for selecting study participants recommended by the World Health Organization (WHO) for producing international growth standards^3^ have not been followed because healthy, uncomplicated pregnancies are rarely studied longitudinally with MRI.

Two-dimensional ultrasound (2D US) images are routinely obtained throughout pregnancy from less than 12 weeks’ gestation. Current US machines produce excellent soft tissue contrast and high-resolution images, and enable acquisition of three-dimensional (3D) volumes. To assess fetal brain maturation, structural US-based metrics have been derived from qualitative scoring of developmental stage^23^ or manual measurement of specific structures^24^. We have mapped US image patterns to gestational age and brain maturation^25,26^, and quantified volumetric growth of cortical^27^ and deep subcortical grey matter^7^ and intracranial tissues^6^. We have also described, mainly in medium- to high-risk pregnancies, five 2D US-derived fetal cranial growth trajectories, associated with differential growth and neurodevelopmental outcomes at 2 years of age, that changed within a 20- to 25-week gestational age window^28^. However, the derivation of a large-scale, international volumetric representation for quantifying normal population variability of the maturing fetal brain from a diverse international data set is lacking.

Therefore, we aimed to construct an atlas of healthy fetal brain maturation derived from US data collected as part of the Fetal Growth Longitudinal Study (FGLS) of the INTERGROWTH-21st Project^2^, which included a postnatal follow-up study to 2 years of age. FGLS has already produced international prescriptive standards based on WHO recommendations^3^, for fetal and neonatal growth^29^, including maturation of the cerebellum and Sylvian fissure^30^ and size charts for five fetal brain structures^31^. Crucially, fetuses in FGLS had satisfactory growth and neurodevelopment at 2 years of age^4,5^, confirming their suitability for constructing growth standards. Combining these prospectively collected, large-scale, international, population-based data with deep learning and advanced image processing tools, we have constructed a four-dimensional (3D + time) parametric map of macroscopic brain anatomy and its temporal in utero evolution, which is presented here.

## Normative fetal brain maturation atlas

**Fig. 1:**
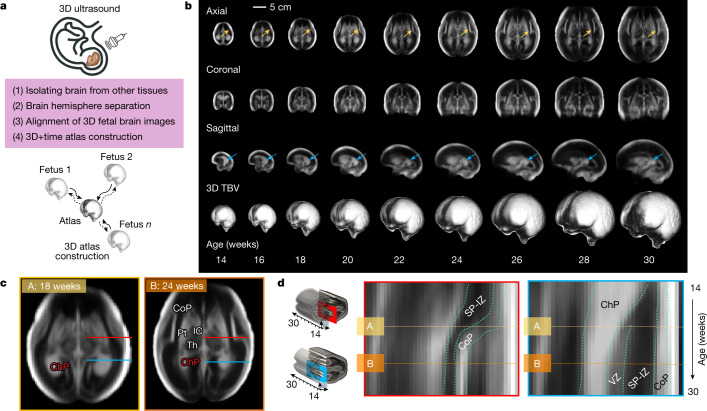
US-derived, normative atlas of the fetal brain with satisfactory growth and neurodevelopment up to 2 years of age. **a**, Summary of image processing steps for atlas construction. A 3D image of the fetal head is collected using an US probe and, after a series of image processing steps (including brain extraction, hemispheric separation and brain alignment), each weekly atlas template is constructed using groupwise registration. **b**, 3D + time atlas templates depicting the fetal brain at even gestational weeks for the axial (top), coronal (middle) and sagittal (bottom) views. **c**, Axial views of the fetal brain at 18 (A) and 24 (B) weeks’ gestation. **d**, Kymograph showing the emergence and thickness changes of laminar tissues of the cerebral mantle at two locations (that is, the two gestational timepoints are marked by horizontal lines A and B). Horizontal lines in **c** correspond to the two cross-sectional locations of the kymograph: red at the level of the thalamus and postcentral gyrus; blue at the level of the ChP. IC, internal capsule; Th. thalamus; Pt. putamen; CB, cerebellum; SP, subplate; IZ, intermediate zone and VZ, ventricular zone (or germinal matrix).

**Extended Data Fig. 1:**
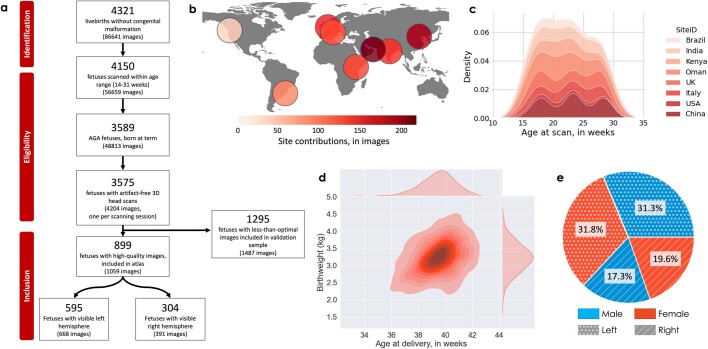
Summary statistics of fetal subjects from the INTERGROWTH-21^st^ FGLS included in the present study. (a) Flowchart summarizing the inclusion criteria, the number of fetuses, and number of scans remaining at each step. (b) World map displaying the contribution of each of the eight countries to the image samples included in the fetal brain atlas. (c) Density plot highlighting the week-by-week contribution of each country. (d) Bivariate Gaussian plot showing the distribution of subjects by birthweight (kg) and gestational age at delivery (weeks). (e) Proportion of scans from male and female fetuses included in the present study, and their contributions to the construction of the left and right cerebral atlases.

Volumetric US image templates were constructed using the pooled data (see the section ‘Morphological variability across sites’) from eight international study sites to represent the human fetal brain at a submillimetre resolution (Fig. 1a; see Methods for image processing steps). The templates, which are equally representative of all brain volumes included (Fig. 1a), characterize weekly stages of brain maturation from 14 to 31 weeks’ gestation (Fig. 1b). To do this, we used 1,059 optimal quality, 3D US volumes from 899 accurately dated fetuses (see ‘Gestational age estimation methodology’ in the Methods) in the cohort that were born at term and appropriately grown (Fig. 1b, Supplementary Video 1 and Extended Data Fig. 1a). The contribution of volumes to the atlas from each site was 3.4% (36/1,059, Seattle), 8.1% (86/1,059, Pelotas), 11.0% (117/1,059, Turin), 11.7% (124/1,059, Nairobi), 12.8% (136/1,059, Nagpur), 14.3% (151/1,059, Oxford), 18.2% (193/1,059, Beijing) and 20.4% (216/1,059, Muscat) (Extended Data Fig. 1b), and all sites were represented in the atlas constructed for each gestational week (Extended Data Fig. 1c). Differences in each site’s contribution to the pooled sample size were due to the availability of high-quality images.

The atlas’ spatial resolution (0.6 × 0.6 × 0.6 mm^3^) enables depiction of cerebral lamination by gestational age, matching with the zonal organization observed in magnetic resonance images and histological sections^32^ (Fig. 1c,d). Of the seven histological compartments observable by MRI during this 17-week period (cortical plate (CoP), marginal zones, ventricular, periventricular, subventricular and intermediate zones and the subplate), four (CoP, ventricular zone and a combination of the intermediate zone and subplate) are distinctly visible in our atlas.

The cell-dense CoP appears as a strongly echogenic smooth layer until 16 weeks’ gestation and then progressively folds with advancing gestational age. From 16 weeks’ gestation, the process of Sylvian fissure operculization is marked by the indentation of the opercula on the ventrolateral surface. The separation between the intermediate zone and subplate is not clearly visible, but their combined hypoechogenic region thickens progressively until 28 weeks’ gestation (Fig. 1d). The ventricular zone is visible as a narrow echogenic region, contouring the anterior and posterior horns of the ventricles. In addition, we observed the interfaces within the basal ganglia: the putamen, caudate nucleus, globus pallidus and internal capsule, from as early as 18 weeks’ gestation, which has not previously been described using US (Fig. 1c).

### Atlas agreement with MRI

**Fig. 2:**
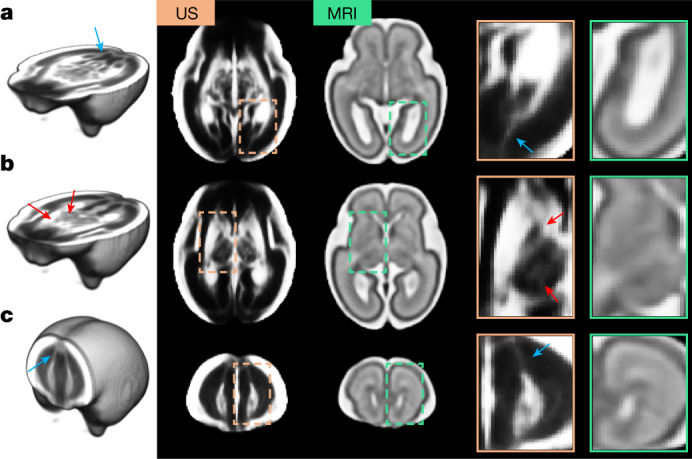
Comparison between US and MRI atlases of the fetal brain. **a**–**c**, Age-matched templates shown at two axial views: level of the ventricles (**a**) and level of the thalami (**b**), and one coronal view (**c**). Structures colocalize in both modalities, but US templates showed sharper tissue boundaries in the subcortical grey matter areas (red arrows). Blue arrows indicate possible white matter fibre bundles in the US atlas, which are not visible in the MRI atlas. Note that the contrast of the US atlas has been edited to highlight the key structures.

Our weekly atlas templates correspond to the structures visible from 21 to 31 weeks’ gestation in the Computational Radiology Laboratory (CRL) MRI-based atlas of human fetal brain maturation^9^. Owing to the unique characteristics of each modality’s physical interactions with brain tissue, a one-to-one intensity mapping between the 3D US and MRI modalities was not possible. Nevertheless, Fig. 2 shows that the tissue boundaries are visible and colocalize in the two modalities, and the shape and spatial extent of the structures are comparable in age-matched templates.

**Extended Data Fig. 2:**
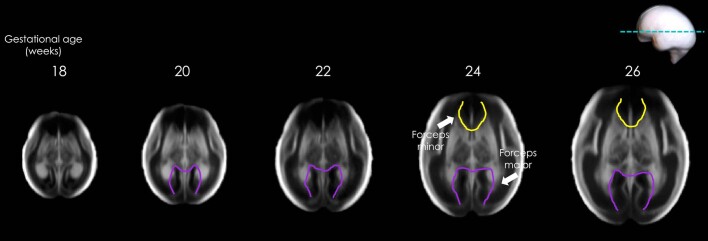
White matter fibre bundles. Evidence of visibility of possible white matter fibre bundles, as described in *Jaimes et al.* (Hum Brain Mapp. 41:3177–3185, 2020) (forceps minor in yellow; forceps major in purple).

Although the MRI atlas shows good separation between the subplate and intermediate zone, some structures are more clearly distinguished in the US versus magnetic resonance images: in particular, the boundaries separating the thalamic nuclei, putamen and internal capsule (Fig. 2b, red arrows) from the posterior lateral ventricle cavity (Fig. 2a). US also identified possible white matter fibre bundles (for example, forceps major from 18 weeks’ and minor from 24 weeks’ gestation, Extended Data Fig. 2) that are usually only visible in diffusion-weighted MRI sequences^33,34^ (Fig. 2a,c, blue arrows).

## Morphological variability across sites

**Fig. 3:**
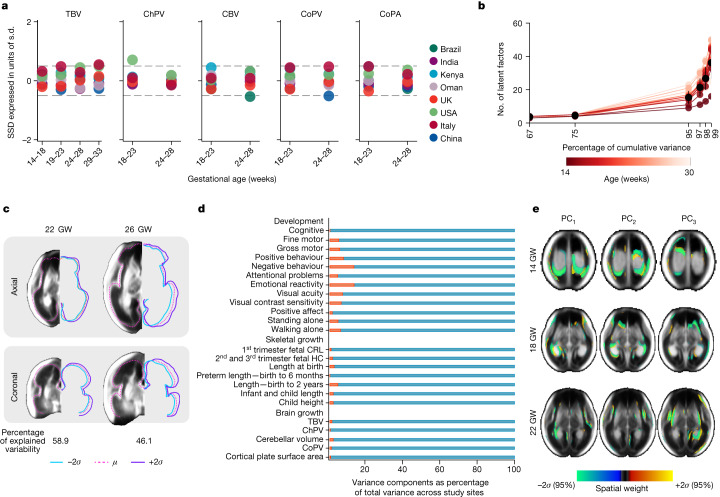
Structural variability among normative fetuses with satisfactory growth and neurodevelopment until 2 years of age. **a**, SSD for TBV (*n* = 1,059, 14–31 weeks’ gestation), ChPV (*n* = 851, 14–31 weeks’ gestation), CBV (*n* = 534, 18–26 weeks’ gestation), CoPV (*n* = 534, 18–26 weeks’ gestation) and CoPA (*n* = 534, 18–26 weeks’ gestation). SSD calculated by: (site mean of the given structure minus all sites’ mean of the same structure)/all sites’ s.d., all values across all gestational ages of the study. **b**, Scree plot showing the structural variability explained against the number of latent factors per gestational week, computed on the basis of PCA and tensor-based morphometry. **c**, Effect of changing the CoP with the first latent factor by +2 s.d. (in purple), mean shape (in dashed pink) and −2 s.d.s (in blue) at 22 and 26 weeks’ gestation, illustrating that size accounts for the most prominent structural differences across the fetal population. **d**, Variance component analysis of developmental, skeletal and brain growth (evaluated in this study). Red bars are the percentage of total variance explained by between-site variability for each growth measure. **e**, Illustrating the regions that explain 60.7, 56.5 and 60.1% of shape variability across the fetal population at 14, 18 and 22 weeks’ gestation, respectively, after size correction. GW, weeks’ gestation and PC, principal component.

To ascertain the validity of pooling the image data from the eight study sites, we used two complementary strategies: variance component analysis and standardized mean site differences^2,5^. Variance component analysis revealed that only between 1.3% and 7.9% of the total variability in total and structural brain volumes could be attributed to between-site differences (Fig. 3a and Supplementary Table 1). Figure 3d shows the comparison between these results and those from FGLS (fetal growth 1.9%, newborn length 3.5%, neurodevelopment at 2 years of age 1.3–9.2% across domains)^2,5^ and the WHO Multicentre Growth Reference Study, which produced the WHO Child Growth Standards (infant length 3%)^35^.

Second, for each site and for each volumetric measure, we calculated the standardized difference between a site mean and the mean of all sites together. The difference was then expressed as a proportion of all sites’ standard deviation (s.d.), that is, the s.d. of the data pooled across all sites for all gestational ages. This resulted in the standardized site difference (SSD), similar to a *z*-score, expressed in units of all sites’ s.d. The SSD allowed for direct comparisons of volumetric measures across study sites, standardized by the corresponding pooled s.d. and adjusted for gestational age (Fig. 3a and Supplementary Tables 9–13). We found that of the 96 possible SSD comparisons for all structures, 92 were within the −0.5 to 0.5 units of s.d., the prespecified interval in the FGLS protocol on the basis of WHO recommendations^3^. Of the four SSDs outside this interval, three were marginally so, that is, total brain volume (TBV, 0.55), cerebellar volume (CBV, −0.54) and CoP volume (CoPV, −0.51); the fourth was the choroid plexus volume (ChPV, 0.71).

As biological growth is a multiplicative process, advancing gestation and variation in genetic and environmental conditions, in addition to methodological and technical issues associated with US scanning, should contribute to increased variability among individuals within a population^21,22^. To define the bounds of structural normality within this healthy fetal cohort, we examined whether any latent factors explain a substantial proportion of the variance within the atlas map of each gestational week, and for a set of volumetric measures. That is, for each gestational week, principal component analysis (PCA) extracted the latent factors (or components) that capture as much of the sample variance observed in the whole set of fetal brain images as possible. Voxel-level analysis of latent factors enables deeper understanding of the spatial and structural diversity, revealing inter-individual differences within a population.

Morphological diversity increased with gestational age (Fig. 3b), as evidenced by the progressively increasing number of components that accounted for more than 95% of cumulative variances at each gestational age, that is, as new structures emerged and grew over time. The population variance maps also summarize the atlas’ representational power (Fig. 3e), defining the structural bounds of healthy brain shape and appearance within an international cohort.

Furthermore, as these inter-individual differences can be indexed by each latent factor, examination of scree plots (Fig. 3b) provided evidence that the first three factors captured the largest share of the variance, with comparatively weaker subsequent factors: a pattern observed across all weeks of gestation. The first latent factor seemed to capture macroscopic size differences across the study population at each week of gestation, which accounted for the largest share (mean 46.9%) of the total structural variance (Fig. 3c). The second (mean 18.4%) and third (mean 9.7%) latent factors indicated localized variance in shape.

On average, fewer than four latent factors were required to describe 1 s.d. (68%) of the study population at all the weeks of gestation, and 15 factors captured 2 s.d. (95%). Inter-fetal size variability accumulated with gestational age for TBV (verified by White’s Lagrangian multiplier test, *χ*^2^ = 29.05, *P* < 0.001) and CPV (*χ*^2^ = 36.51, *P* < 0.001). Although variances visibly increased in CoPV, CoP surface area (CoPA) and CBV growth, these did not pass multiple comparison correction tests.

To better understand the region-specific variation in structural morphology, we excluded the effect of the first factor by normalizing the images by TBV through performing the PCA on the deformation fields after the application of global linear transformation (affine registration) in the atlas construction pipeline. Colour-coded maps reveal the brain voxels that corresponded to the maximum factor scores (Fig. 3e), and the regions with the highest inter-individual morphological variations detected by the respective latent factor. Colours correspond to the spatial extent (or spatial weight) to which specific structures occupy the brain (relative to the population mean).

Spatial weights are shown to represent the 2 s.d. ($\pm 2\sigma$) of shape variations. For each week of gestation, positive values (warm shades) indicate regions where there may be participants with larger than-average structures and the opposite is true for negative values (cool shades). At 14 weeks’ gestation, inter-individual variability was localized in the ChP (choroid plexus) (Fig. 3e), with the greatest contribution coming from symmetrical differences in the posterior region. The most pronounced differences, with some asymmetry, were found from 18 weeks’ gestation in the insula and lateral sulcus (Fig. 3e), which are part of the prelanguage network. From 22 weeks’ gestation, variability was also detected in the temporal superior sulcus, middle occipital gyrus, calcarine sulcus and precuneus. Shape differences in subcortical grey matter structures (putamen and internal capsule) contributed to the 13.05% population variability seen at this gestational age. In summary, pooling data across sites is supported by the very low proportional contribution of between-site variance to the total variance and by the very low standardized site mean discrepancies across gestational ages and brain structures.

## Spatiotemporal maturational schedule

**Fig. 4:**
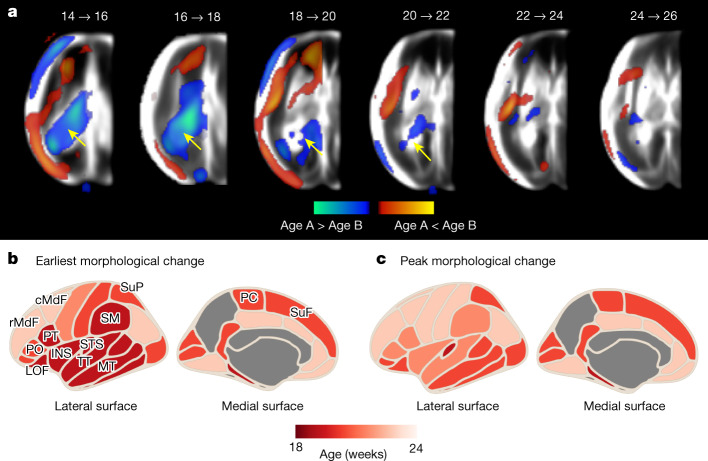
Timing of the spatiotemporal changes among normative fetuses with satisfactory growth and neurodevelopment until 2 years of age. **a**, Significant regions derived by the FSL RANDOMISE non-parametric permutation test (family-wise error-corrected, *P* < 0.05), overlaid on the US atlas templates for each 2-week gestational age interval. ChP shrinkage is indicated by the yellow arrow. **b**,**c**, Age and cortical regions at which morphological changes were first detected (**b**) or showed peak morphological change (**c**). Cortical surface maps were created using the Python-based ggseg package^48^. The parcellation-based results do not apply before 18 weeks’ gestation.

**Extended Data Fig. 3:**
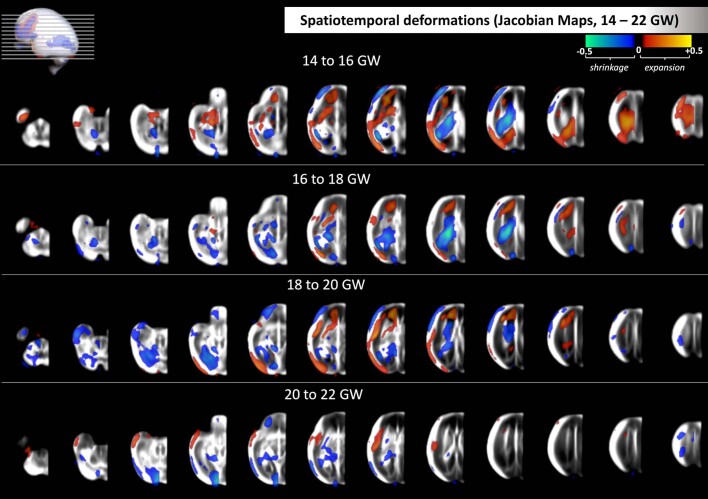
Spatial deformation maps from 14 to 22 weeks’ gestation. The Jacobian maps highlight the regions undergoing expansion (warm shades) or shrinkage (cool shades) within each two-week interval between 14- and 22-weeks’ gestation. Deformation maps are calculated as the difference between the mean log-Jacobian maps of each age group). This figure complements the single-slice views shown in Fig. 4 of the main text.

**Extended Data Fig. 4:**
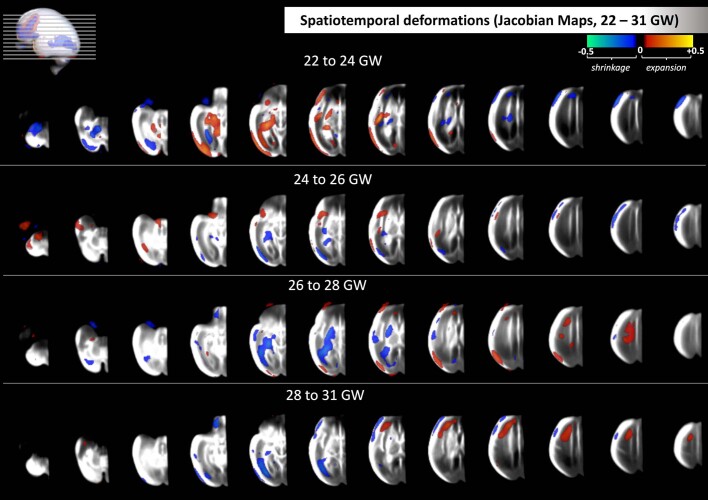
Spatial deformation maps from 22 to 31 weeks’ gestation. The Jacobian maps highlight the regions undergoing expansion (warm shades) or shrinkage (cool shades) within each two-week interval between 22- and 31-weeks’ gestation. Deformation maps are calculated as the difference between the mean log-Jacobian maps of each age group). This figure complements the single-slice views shown in Fig. 4 of the main text.

To show the dynamic process of healthy maturation, we calculated the time-specificity for each brain region of interest, across 2-week gestational age windows (temporal changes in Fig. 4a and Extended Data Figs. 3 and 4). By removing global size differences through size-corrected 3D deformation fields (by first removing isotropic scaling effects), we were able to highlight patterns of local dilation and contraction. Heterogeneous patterns were observed throughout the 17-week period. From 14 to 19 weeks’ gestation, macrostructural dynamic changes in brain anatomy were observed including, most of all, ChPV shrinkage (Fig. 4a).

From 20 to 31 weeks’ gestation, the most notable morphological changes were consistently observed in the insular cortex (INS) and peri-Sylvian regions, both associated with language development. For each subset of scans within a 2-week gestational age window, we extracted the percentage of voxels undergoing notable morphological change. By inspecting 34 cortical areas (delimited by the Desikan–Killiany atlas^36^), for which parcellation maps separating the cortical regions were available from 20 to 31 weeks’ gestation, we demonstrated regional variability in the schedule of folding trajectories.

Out of the 34 cortical areas studied from 20 weeks’ gestation, all but the fusiform gyrus showed a significant gestational age effect (Fig. 4b). Between 20 and 22 weeks’ gestation, the gyri within the INS, temporal lobe (middle temporal gyrus), Broca’s (pars opercularis) and Wernicke’s (superior temporal gyrus, Heschl’s gyrus, supramarginal gyrus and bank of the superior temporal sulcus) areas progressively expanded.

INS expansion persisted until 26 weeks’ gestation, rostrally extending to include regions that will develop by 24 weeks’ gestation as Broca’s area (pars triangularis, pars orbitalis), which is associated with speech and language processing. In the parietal lobe, the paracentral and postcentral gyri deformed between 22 and 24 weeks’ gestation and were soon followed by the precentral gyrus between 24 and 26 weeks’ gestation.

Active folding of the frontal lobe was observed between 26 and 28 weeks’ gestation, with significant changes detected in the middle frontal, superior frontal, frontal pole and orbitofrontal gyri. These findings accord with the general ventral to dorsal pattern of cortical maturation in the second trimester shown in Fig. 4b. However, the timing is more detailed than the spatiotemporal schedule previously described in human fetuses^1,9,10,14^ and non-human primate fetuses^37^ at comparable gestational ages because of the rigorous methodology used to estimate gestational age accurately (see ‘Participants and fetal US scans’ in the Methods). Furthermore, the progressive expansion of the CoP, we detected from 18 weeks’ gestation, is compatible with operculization in the second trimester^23,38^.

## Spatiotemporal sex similarities

The mean gestational ages at which 3D volumes were acquired for male and female fetuses were 21.7 ± 4.4 and 22.5 ± 4.4 weeks, respectively, and the gestational age distributions did not significantly differ (Kolmogorov–Smirnov test, *D*_M_ = 514, *D*_F_ = 544 = 0.08, *P* = 0.061, two-tailed). When adjusted for well-recognized brain size differences^39^ (Supplementary Table 2), voxel-wise analyses revealed no statistically significant structural differences between sexes. Specifically, no voxels on the cerebral cortex survived multiple comparisons testing for asymmetry, which suggests that sexual dimorphisms have not yet manifested before 31 weeks’ gestation.

## Spatiotemporal asymmetry

**Fig. 5:**
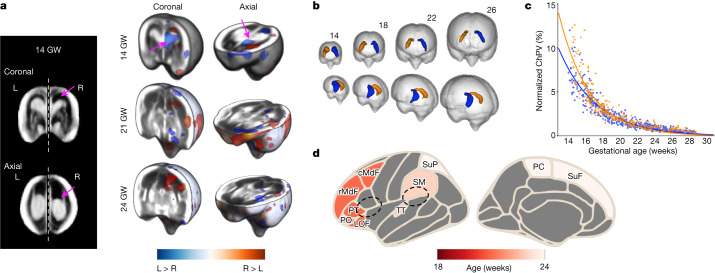
Earliest detection of structural fetal brain hemispheric asymmetries. **a**, 3D reconstructions at 14, 21 and 24 weeks’ gestation with the log-Jacobian maps overlayed on the US atlas template. Blue indicates regions where the left hemisphere is larger than the right (L > R; pink arrows) and vice versa (R > L). **b**, Physical configuration of the fetal ChP, shown at 4 week intervals starting from 14 weeks’ gestation. Average ChP shape for the left (blue) and right (orange) hemispheres are shown separately. **c**, Normalized ChPV, highlighting the rate of ChP shrinkage relative to TBV. **d**, Visual summary of the population average of cortical asymmetries. Colours indicate the gestational age at which asymmetry was first detected. Dashed lines indicate Broca’s and Wernicke’s areas. The parcellation-based results do not apply before 18 weeks’ gestation.

We detected emergent asymmetries by calculating average volumetric differences and voxel-wise analysis for each week of gestation. Cohort-level meta-analysis reveals, to our knowledge, the earliest in vivo report of the fetal brain’s asymmetrical organization and differential growth rates. Specifically, we found structural size differences between the two cerebral hemispheres, with a larger ChPV in the left hemisphere from 14 weeks’ gestation (*z* = −2.942, *P* = 0.003; Fig. 5a,c). Left-dominating ChPV asymmetry persisted throughout the second trimester, but increasing gestational age was associated with progressively less asymmetry (*z* = −11.04, *P* < 0.001). No hemispheric differences were found in TBV (*z* = −0.229, *P* = 0.819; CoPV (*z* = +1.086, *P* = 0.278) or CoPA (*z* = +0.108, *P* = 0.914).

**Extended Data Fig. 5:**
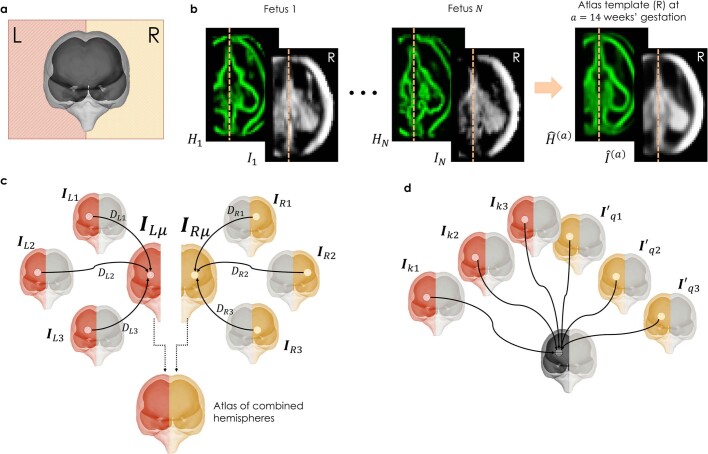
Image data preprocessing for atlas construction and statistical analysis. The image processing steps are shown only for the right hemisphere, but the same process was carried out for the left hemisphere. (a) Individual US scans were separated into left (L) and right (R) cerebral hemispheres, and only the hemisphere distal to the US probe was kept for subsequent analysis. The image processing steps are shown only for the right hemisphere but the same process was carried out for the left hemisphere. (b) Examples of brain axial slices from two individuals at 14 weeks’ gestation (in grayscale), and the edge map used to enhance features in the atlas construction step (in green). The resulting atlas template is shown on the right-hand side. (c) Separate atlas templates were constructed for each cerebral hemisphere using groupwise multi-channel registration, and combined for visualization purposes. Arrows represent diffeomorphic mapping between the atlas and each individual image. (d) Illustration of image orientation for statistical analysis (e.g. tensor-based volumetry to detect regions of asymmetry or temporal change). For asymmetry analysis, $k=L$ and $q=R$ in reference to the mirrored right hemispheres. For temporal change analysis, $k=a$ and $q=a+2$, in reference to the gestational ages, in weeks, at the start ($a$) and end ($a+2$) of the interval.

A cohort-level morphological asymmetry index was calculated to determine the direction of regional asymmetry. This was achieved by contrasting the means of the left- and (mirrored) right-sided deformation fields that spatially transform the brain hemispheres into a common atlas template (Extended Data Fig. 5) enabling explicit detection of gestational age-specific asymmetries. We applied the Kolmogorov–Smirnov non-parametric statistical test to the deformation fields (see Methods for details), expressed as morphological asymmetry index to capture the nonlinear effects of hemispheric asymmetry at the voxel level (Fig. 5a). Asymmetric regions are shown on the Desikan–Killiany atlas^36^ (Fig. 5d).

Overall, the maturing fetal brain does not show bilateral symmetry. Although the sampled left and right hemispheres differed in scale (Kolmogorov–Smirnov test *D*_L_ = 399, *D*_R_= 659 = 0.168, *P* < 0.001), the analysis of size-corrected brain volumes showed, rather, that there are regional asymmetries emerging at different stages of maturation. For example, across the 34 cortical regions available from 21 weeks’ gestation, the earliest asymmetries were detectable at 21 weeks’ gestation in the middle frontal gyrus, inferior frontal gyrus (pars orbitalis and triangularis) and lateral orbitofrontal gyrus (Fig. 5d). By 23 weeks’ gestation, the supramarginal and transverse temporal (Heschl’s) gyri were distinctly larger on the right hemisphere, and further asymmetries were detectable in the paracentral and superior parietal gyri at 24 weeks’ gestation. Three of these regions will eventually form Broca’s area (inferior frontal gyrus) and Wernicke’s areas (supramarginal and Heschl’s gyri), constituting the primary auditory cortex and the brain’s language centre. With the exception of the triangular part of the inferior frontal gyrus, all asymmetries detected on the frontal, temporal and parietal lobes of the cerebral mantle showed a rightwards dominance (R > L).

## Fetal brain growth patterns

**Fig. 6:**
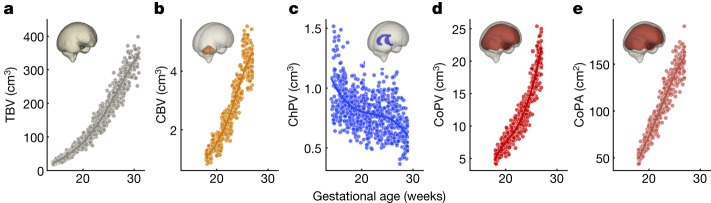
Distribution of fetal brain growth measures. **a**–**e**, Normative fetal trajectories for TBV (**a**), CBV (**b**), ChPV (**c**), CoPV (**d**) and CoPA (**e**).

**Extended Data Fig. 6:**
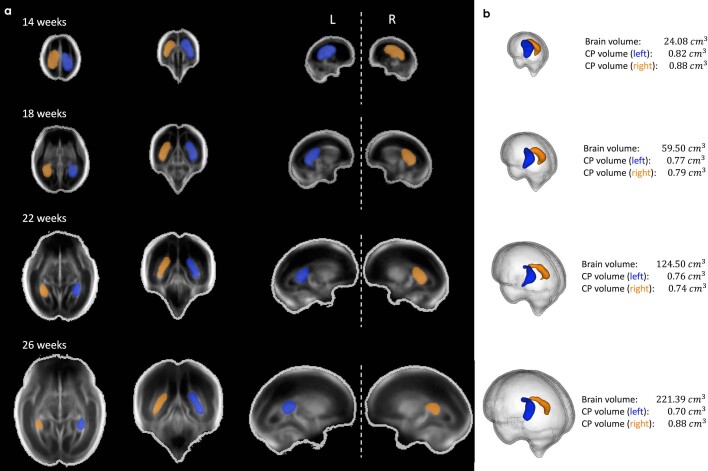
Choroid plexus asymmetry. Mean total brain volume, left and right choroid plexus volumes derived from the fetal brain atlas at four gestational timepoints. (a) Axial (left column), coronal (middle), and sagittal (right) views shown, overlayed with segmentation maps of the choroid plexus (left hemisphere shown in blue; right shown in yellow). (b) 3D rendering of choroid plexus segmentations, relative to total brain volume.

Overall, the patterns of fetal brain growth show that TBV increased monotonically 13-fold between 14 and 31 weeks’ gestation (from a mean of 24.08 to 317.60 mm^3^; Fig. 6a), well aligned with the 50th centile of the INTERGROWTH-21st head circumference standard^29^ with a strong positive correlation between the two measures (*r* = 0.975; *P* < 0.001). CBV also followed a near-linear increase in agreement with previous reports based on 2D linear measurements^30^. CoPA and CoPV both increased with gestational age, with volume following a quadratic pattern (*P* = 0.004, Fig. 6d,e) that maintained a steady rate of growth, consistent with previous MRI reports^15,21^. There was also pronounced and consistent ChPV regression (average 0.82 to 0.7 mm^3^) (Fig. 5c and Extended Data Fig. 6), relative to TBV. At 14 weeks’ gestation, the telencephalon was largely occupied by the ChP, representing 8.3% of TBV. By 30 weeks’ gestation, the ChP represented a small structure inside the lateral ventricle (*z* = −11.0, *P* < 0.001), occupying roughly only 0.4% of TBV. This relative reduction in volume was localized within the lateral ventricles and outpaced by a quadratically increasing TBV. The shrinkage occurred in tandem with thickening of the neighbouring subplate-intermediate zone layer (Fig. 1d).

The absolute volumetric measures were separately extracted from each cerebral hemisphere. We found no statistically significant gestational age–hemisphere interactions in any of the parameters, which suggests that the difference between the left and right hemispheres remained constant throughout the second trimester; that is, the hemispheres are growing at a similar rate.

## Validation sample

**Extended Data Fig. 7:**
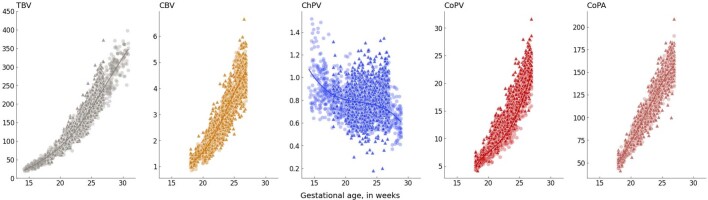
Growth trajectories for total brain volume (TBV), cerebellar volume (CBV), choroid plexus volume (ChPV), cortical plate volume (CoPV), and cortical surface area (CoPA). For each structure, the raw data points are shown for the within-sample data (circles; used for atlas construction), and the out-of-sample data (triangles; used for validation), plotted against gestational age (x-axis). The y-axes are scaled to the units of the corresponding volumetric measure ($c{m}^{3}$ for volume, $c{m}^{2}$ for surface area). Growth trajectories of the mean (solid line) are also shown.

To explore the reliability and validity of the patterns of fetal brain growth presented here, we computed the volumetric measurements of ‘out-of-sample’ brain volumes that were excluded from the sample used for atlas construction because of moderately optimal image quality. A total of 1,487 scans (*n* = 1,295 separate fetuses, also born at term and with equally satisfactory growth and neurodevelopment from early pregnancy to 2 years of age) were preprocessed using the same pipeline (see Methods for details). Volumetric measurements were closely correlated with those derived from the atlas set (Extended Data Fig. 7). The contribution of volumes to the validation sample from each site was 5.4% (81/1,487, Seattle), 8.6% (128/1,487, Nairobi), 11.7% (174/1,487, Turin), 13.0% (193/1,487, Pelotas), 13.2% (196/1,487, Nagpur), 14.3% (213/1,487, Oxford), 15.5% (230/1,487, Beijing) and 18.3% (272/1,487, Muscat).

Having detected the most prominent regional asymmetries at three gestational timepoints (14, 21 and 23 weeks), we examined whether the same patterns appeared in the out-of-sample dataset to illustrate the generalizability and biological significance of our findings. At 14 weeks’ gestation, ChP asymmetry was detected (Welch’s test *D* = 0.413, *P* < 0.001). Similarly, asymmetries were detected in the peri-Sylvian regions. Notably, asymmetry of the superior temporal gyrus, located within Wernicke’s area and associated with auditory processing, was also detected at 21 weeks’ gestation in this fetal cohort: at least 2 weeks earlier than previously reported^18,19^.

## Two-year infant follow-up data

**Extended Data Fig. 8:**
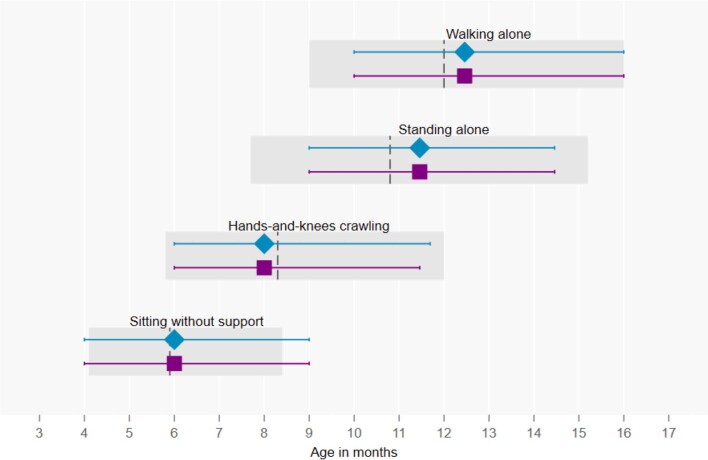
Median age of achievement (3^rd^ and 97^th^ centiles) of four gross motor development milestones. Data are for infants who were included in the present study (blue) and those who were included in the INTERGROWTH-21^st^ Fetal Growth Standards (purple). For comparison, the 3^rd^ and 97^th^ percentiles of the World Health Organization windows of achievement for the same milestones are presented in grey (with the median shown as a vertical line).

At 2 years of age, the anthropometric measures and morbidity rates of the eligible infants seen at follow-up (optimal quality images *n* = 671 out of 899, 74.6%; moderately optimal quality images *n* = 929 out of 1,294, 71.8%) were similar to those from the total FGLS population (*n* = 3,042 out of 4,501, 70.4%) (Supplementary Table 3). The median age at which the infants achieved the four previously used gross motor development milestones (Extended Data Fig. 8) was virtually identical to the total FGLS population^5^, both well within the range of the WHO standards.

The available standardized cognitive, language, fine motor, negative behaviour and positive behaviour scores of the study sample were within the respective centile ranges of the international INTER-NDA (INTERGROWTH-21st Neurodevelopmental Assessment) standards for healthy neurodevelopment at 2 years of age^40^. Only 1.1% of infants had visual acuity and 2.6% contrast sensitivity scores below the norms for 2-year-olds (logMar 0.4 to 0.1 for visual acuity, and 33.3–100% for contrast sensitivity), compared with the low prevalence (0.9 and 2.0%, respectively) reported for the total FGLS population. These data confirm that the study sample used for the construction of the brain atlas and the other measures reported here were adequate for the representation of normative fetal patterns.

## Discussion

This is the largest study, to our knowledge, depicting normative early human brain development, based on 3D US scans performed across eight geographically diverse study sites in a cohort of healthy fetuses, born at term, appropriately grown, with satisfactory growth and neurodevelopment at 2 years of age^4,5^. Our reconstructions of the fetal brain provide the first in vivo depiction, to our knowledge, of the whole second trimester at a level observable with US. This is the critical time of active cell proliferation, migration and synaptogenesis, as well as macroscopic changes. Each 3D template represents the brain’s detailed structural anatomy by gestational age, starting 6 weeks earlier in pregnancy than previous reports^18,20^.

Our work is unique because we: (1) studied a large international cohort of low-risk pregnancies with healthy fetuses that had similar characteristics, including normative health, growth and neurodevelopment outcomes at 2 years of age^4,5^, to those that contributed to the INTERGROWTH-21st prescriptive standards^29,30^; (2) enrolled women with reliable menstrual dates and a confirmed gestational age in the first trimester and scanned them using standardized US equipment and protocols^29^, enabling precise determination of spatiotemporal patterns in fetal brain maturation; (3) described the variability in normative fetal brain development using an advanced image analysis framework and novel, deep learning-based tools; (4) adopted a template-free approach to reduce the bias introduced by selecting a fixed reference fetal brain image; (5) captured in vivo maturation between 14 and 20 weeks’ gestation, filling a 6 week gap in our understanding of early fetal brain development; (6) characterized structural changes and found significant early asymmetries in all three cortical regions delimited by the Desikan–Killiany atlas^36^ that are associated with language development and (7) identified possible axonal fibre tracts only previously observed with diffusion-weighted MRI^34^. Our multinational data were not stratified by variables such as socioeconomic status as these are already accounted for by selecting samples living in adequate environmental conditions and at low risk of adverse outcomes across all study sites. Furthermore, the timing of brain maturation did not vary significantly among sites, which themselves have highly heterogeneous underlying populations that are the products of centuries of migration.

Asymmetry of structure and function is a common feature of biological systems, as found in left hemispheric regions involved in speech production, such as the planum temporale: an extension of Wernicke’s posterior receptive language area, that is ten times larger in adult humans than its right-sided counterpart^41^. Such asymmetry may reflect genetic differences in the control of each hemisphere’s development before neuronal migration is complete^19^. Our results align with asymmetry findings in adulthood for language^42^.

We identified several fetal brain regions that are asymmetric (in terms of volume and structural morphology) that have not previously been described. We have also described a more granular (week-by-week) timing of the emergence of these asymmetries than previous reports^19,20^. The most distinctive region to show asymmetry in our study was the superior frontal, which is formed by the relative overgrowth of the frontal and temporal lobes over the INS associated with very different transcriptomic profiles in the associated cortices^43^.

Our more detailed observations are important because of the limited, sometimes contradictory information available on fetal brain asymmetry from in vivo imaging studies^44^, and methodological limitations in construction of normative data, hence heterogeneity of the results. For example, some studies report earlier cortical folding in the right hemisphere^18–20^, with the right superior temporal sulcus appearing by 23–24 weeks’ gestation^18,19^. In other studies, the left hemisphere has seemed larger, and regions of the frontal and parietal lobes have shown leftwards dominance^45^. Furthermore, most previous reports have focused on maturation from mid to late gestation to later life. We confirmed, however, that the cerebral hemispheres follow separate maturational programmes, with greater rightwards dominance in regions that will develop into Broca’s and Wernicke’s areas, suggestive of prioritization of language readiness.

Our study, therefore, complements and extends understanding of the early origins of structural inter-hemispheric asymmetries, and defines the week-by-week timeline of their emergence with data on satisfactory neurodevelopment at 2 years of age. Having identified ChP asymmetries from 14 weeks’ gestation, we conclude that cerebral lateralization is detectable 6 weeks earlier than previous reports that have relied on smaller, more age-restricted fetal populations^18^. Our measures of volumetric growth were consistent with previous MRI findings^9,46^. We did not identify any statistically significant, region-specific, morphological differences between male and female fetuses, in keeping with a previous MRI study^21^.

**Extended Data Fig. 9:**
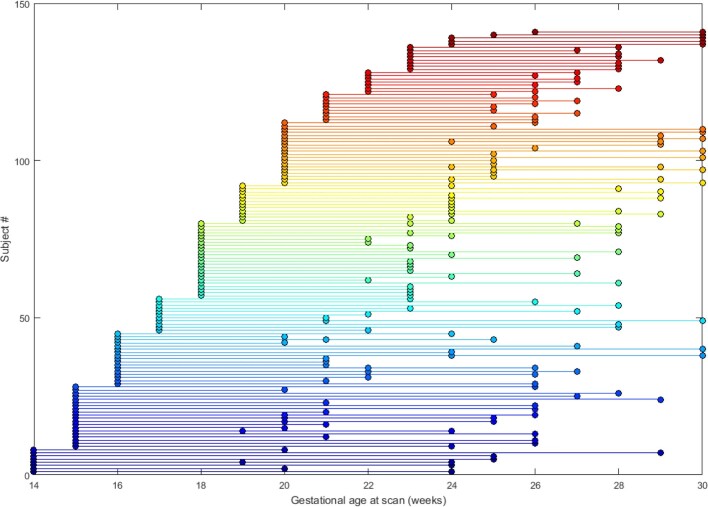
Number of subjects with longitudinal scans. Of the 899 subjects included in the atlas, 141 of them (15.7%) were included more than once (but always at a different gestational week). None of the subjects was included in successive weeks (separated by approx. 4-5 weeks).

Of the 4,321 fetuses that contributed to the INTERGROWTH-21st fetal growth standards^29^ 3,575 (82.7%) had artefact-free 3D US scans and were born appropriately grown at term; of these, only 899 (25.1%) were included in the brain atlas (Extended Data Fig. 1a) because of the very strict eligibility criteria relating to US image quality to ensure optimal visualization of brain structures. This was a technical requirement because atlas construction and image segmentation rely on high-definition visibility of tissue boundaries; however, no selection bias related to a pregnancy-associated condition is likely. Of the 899 participants included in the atlas, 141 (15.7%) were included more than once (but always at a different gestational week). None of the participants was included in successive weeks (separated by roughly 4–5 weeks; Extended Data Fig. 9).

When we examined the 1,487 volumes from the 1,295 (36.2%) eligible fetuses that did not meet the strict image quality criteria, our algorithms estimated sizes that were similar to those derived from the atlas (Extended Data Fig. 5). In addition, the maternal and perinatal characteristics of the pregnancies included in the present study, as well as the infant health outcomes at 2 years of age, were similar to the total FGLS population (Supplementary Tables 4 and 5), although INTER-NDA data were available from only five of the eight study sites. Last, the difficulty of visualizing both cerebral hemispheres with US, especially after 31 weeks’ gestation, due to acoustic shadows and reverberation artefacts as the fetal skull calcifies, meant that, at any given gestational age, we analysed only the distal hemisphere. Thus, although cross-sectional data were used to construct the atlas from our original longitudinal study, we were still able to identify developmental asymmetry within this healthy cohort.

In summary, our atlas provides unique insights into the spatiotemporal patterns of brain maturation in a normative fetal cohort that had satisfactory growth and neurodevelopment from the first trimester of pregnancy up to 2 years of age. We have characterized those processes before 20 weeks’ gestation. Our atlas will be useful as a research tool to investigate the fetal origins of neurodevelopmental disorders^47^. By helping to characterize the extent of deviation from healthy structural maturation and lateralization at critical times during pregnancy, our work will allow more detailed investigation into factors that modify fetal brain maturation and can affect cognitive function in childhood^28^.

## Methods

### Participants and fetal US scans

Three-dimensional US scans of the fetal head were acquired as part of the FGLS of the INTERGROWTH-21st Project^29^ to explore their use for undertaking 2D measurements, and for further exploratory analyses^30,31^. A detailed description of FGLS and its inclusion criteria has been published elsewhere^2^.

In brief, INTERGROWTH-21st was a population-based project, conducted between 2009 and 2016 in eight delimited urban areas: Pelotas (Brazil), Turin (Italy), Muscat (Oman), Oxford (UK), Seattle (USA), Shunyi County in Beijing (China), the central area of Nagpur (India) and the Parklands suburb of Nairobi (Kenya). Participating women, who initiated antenatal care before 14 weeks’ gestation, were selected on the basis of WHO criteria for optimal health, nutrition, education and socioeconomic status needed to construct international growth standards^3^. Hence, they had low-risk pregnancies that fulfilled well-defined and strict inclusion criteria at both population and individual levels^2^.

The last menstrual period (LMP) was used to calculate gestational age (±7 days) provided that: (1) the date was certain; (2) the woman had a usual 24–32 day menstrual cycle; (3) she had not been using hormonal contraception or breastfeeding in the preceding 2 months and (4) any discrepancy between the gestational ages on the basis of LMP and crown–rump length, measured by US at 9^+0^ to 13^+6^ weeks(^+days^) from the LMP was fewer than or equal to 7 days, using the formula described by Robinson and Fleming^49^. To ensure that crown–rump length measures were interpreted consistently, the Robinson–Fleming formula was loaded into all the study US machines; whenever another machine had to be used locally for crown–rump length measurement, a conversion table extracted from the same formula was provided. The crown–rump length technique was also standardized across sites and all ultrasonographers were trained uniformly.

US scans were then performed every 5 ± 1 weeks from 14^+0^ weeks’ gestation to delivery (that is, 14–18, 19–23, 24–28, 29–33, 34–38 and 39–42 weeks’ gestation). Dedicated sonographers performed the US scans using identical, commercially available, equipment (Philips HD-9, Philips Ultrasound), with a curvilinear abdominal 3D transducer (V7-3). A transvaginal probe was not used because it would have been culturally inappropriate in many settings. The US probe was positioned such that the central axial view was collected at the level of the thalami, and the angle of insonation was adjusted to include the entire skull (roughly 70°) for a typical volume acquisition time of 4 s. We conducted centralized hands-on training of sonographers, and the Oxford-based Ultrasound Quality Control Unit regularly carried out site-specific standardization procedures to ensure proper use of the US equipment and protocol adherence.

Extended Data Fig. 1a presents a flowchart of the data inclusion pipeline used to generate the 4D (3D + time) probabilistic atlas based on 899 healthy singleton fetuses in the FGLS database that were appropriately grown and born at term (451 (50.2%) females and 448 (49.8%) males). The maternal and perinatal characteristics of this subpopulation were similar to the total FGLS population (*n* = 4,321) (Supplementary Tables 3–5 and Extended Data Fig. 8).

### Infant follow-up

Across all sites, standardized clinical care and feeding practices were implemented using the INTERGROWTH-21st Neonatal Group protocols (www.intergrowth21.org.uk). Exclusive breastfeeding up to 6 months and appropriate nutritional support for infants born preterm were promoted during and after pregnancy. Detailed information was obtained from the mother at the age of 1 and 2 years about the infant’s health, severe morbidities, hospitalizations, length of breastfeeding, timing of the introduction of solid food, age at weaning, feeding practices and food intake, using specially produced forms (www.intergrowth21.org.uk). The proportion of infants receiving breast milk, and vitamin and mineral supplements, and those following a special diet were estimated at the ages of 1 and 2. Similarly, at age 1 and 2, the infant’s weight, length and head circumference were measured following WHO protocols^50^, and their age- and sex-specific *z*-scores and centiles were compared to the WHO Child Growth Standards^35^. These anthropometric measures, as indicators of general nutrition at the age of 2, are strongly predictive of later attained height, development and human capital^51^.

### Neurodevelopment assessment

We assessed neurodevelopment at 2 years of age using the INTER-NDA (www.inter-nda.com), an international, psychometrically valid and reliable, standardized tool, targeted at children 22–30 months of age, which measures several dimensions of early development using a combination of directly administered, concurrently observed and caregiver reported items^52^. It was designed to be implemented by non-specialists across multinational settings, and includes a reduced number of culture-specific items comprising six domains measuring cognition, language, fine and gross motor skills, and positive and negative behaviour, in an assessment time of 15 min on average.

The INTER-NDA has been validated against the Bayley Scales of Infant Development III edition^53^. On the basis of established guidelines^54^, it showed ‘good’ agreement with interclass coefficient correlations across domains ranging between 0.75 and 0.88. Its norms are the first international standards of early child development, constructed according to the prescriptive WHO approach using data from five of the eight INTERGROWTH-21st study sites^40^. So far, more than 18,000 children in 22 countries have been assessed using the INTER-NDA.

Attentional problems and emotional reactivity were measured on the respective subscales of the Preschool Child Behavior Checklist^55^; responses were based on caregiver reports. Vision was assessed using the Cardiff Visual Acuity and Contrast Sensitivity tests for binocular vision^56^. These are indicative of the integrity of the central visual pathway, and as directly observed neurodevelopmental markers, are unlikely to be affected by cultural influences and co-occurring disturbances in cognitive, hearing and language skills.

Motor development was assessed against four WHO milestones that are less likely to be affected by recall bias: sitting without support, hands knees crawling, standing alone and walking alone^57^. Trained staff collected the data on a form with pictures of the relevant child positions and corresponding definitions. Parents were asked to report the age in months and weeks when they ‘first observed’ or ‘never observed’ the milestones. We assessed the age (in months) at which WHO gross motor milestones were first achieved.

All INTER-NDA assessors were trained and standardized centrally. All assessors were subject to a protocol adherence and reliability assessment following training; only those with protocol adherence scores in excess of 90% and inter-rater reliability of more than 0.8 conducted assessments. The administration of the above tests was supported by a tablet-based data collection and management system. Field staff were unaware of the INTER-NDA domain and total scores for individual children and sites. Data were uploaded onto secure servers as soon as each assessment was completed and compared to the international normative values established by the INTERGROWTH-21st Project^40^.

### Image curation

Volumetric images were selected from the INTERGROWTH-21st database on the basis of image quality criteria alone and AILN was blinded to the study site information during image selection. Briefly, inclusion criteria required the fetal head to occupy at least 30% of an artefact-free image (defined as free from the aberrations introduced by spontaneous fetal movement during scanning, acoustic shadows caused by the skull’s convex shape or reverberations from structures in the proximal hemisphere or maternal tissues), and clear visibility of structures in the distal hemisphere, for example, thalami, CoP, Sylvian fissure and ChP (details in Supplementary Table 6).

To detect artefact-free cases to achieve the best quality atlas, AILN manually assessed all 48,813 3D volumes; each volume took 1–2 min to assess depending on the overall quality, that is, a total of roughly 1,200 h. Artefacts included fetal motion, strong acoustic shadows and poor contrast, which limited visualization of brain structures in the distal hemisphere. The final selection of 899 cases depended on image quality, which was further assessed on the basis of the criteria provided in Supplementary Table 6 after the initial screening. Each assessment took on average 3 min (rough range 1–6 min), that is a total of roughly 210 h. We did not have access to a tool to automate the process.

We ultimately excluded more than 55,000 scans obtained between 14^+0^ and 30^+6^ weeks’ gestation on the basis of image quality (Supplementary Table 6 and Extended Data Fig. 1a). Extended Data Fig. 1c shows the distribution of US scans across these gestational ages for each study site.

As a result of acoustic shadows and reverberation artefacts, only the distal cerebral hemisphere typically contains clearly discernible brain structures in US images. All analyses were, therefore, performed separately for images presenting the left and right hemispheres in the distal region of the US scan, resulting in two atlas (template) maps per gestational week. Each hemispheric map was constructed from a minimum of ten US scans per gestational week (Supplementary Table 7).

### Image preprocessing

Each individual US image was processed following a series of manual and automated procedures. As summarized in Fig. 1a, these mainly included: (1) rigid alignment to a standardized coordinate space, (2) brain extraction and (3) structural enhancement. Before entering this pipeline, the 3D volumes were resampled to an isotropic voxel size of 0.6 × 0.6 × 0.6 mm^3^ using trilinear interpolation, from a median size of 0.32 × 0.51 × 0.85 mm^3^. To ensure that all fetal brains were included whole, the images were cropped to 160 × 160 × 160 voxels around the centre of the brain.

#### Rigid alignment

Fetal brains show inter-participant variability in shape, size and localization. In US images, the position of the US probe relative to the head directly affects the orientation of the imaged brain. Establishing the structural coordinate system is crucial as it reduces the degrees of freedom in the non-rigid transformations required for atlas construction. To compare anatomy across participants, we performed semi-automated brain alignment in two steps to bring the brains into structural correspondence. First, a deep learning-based, convolutional neural network was used to localize the brain within the 3D volume, exclude maternal and extracranial tissues, and linearly align each brain to a common 3D coordinate space^8^. A secondary manual correction step ensured inter-participant co-alignment across all images by following the convention of the stereotaxic space of the MNI-Colin27 template^58^. The cropped brain images were rigidly aligned to a standardized 3D coordinate space, using a seven-parameter linear transformation (three translation terms, three rotation terms, one scale term to preserve the aspect ratio). This was achieved using MATLAB’s graphical user interface toolkit (MathWorks, 2021), which involved locating the midsagittal plane (that coincides with the longitudinal fissure separating the two cerebral hemispheres), and aligning key neuroanatomical landmarks therein (procedure shown in Supplementary Video 2). Once aligned, it was easy to determine the left and right hemispheres. We found that manually locating three landmark points on the corpus callosum and cavum septum pellucidum complex and rigidly registering these to a fixed point-based template achieved sufficient structural alignment across all images within a specific gestational age (Supplementary Video 2).

### Fetal brain masking

Extracranial tissues (for example, eye orbits, scalp, maternal tissues) were removed using brain masks from the CRL MRI brain atlas constructed from 81 fetuses scanned between 19 and 39 weeks’ gestation^9^. We manually registered the CRL atlas template of the corresponding gestational week to each aligned US image, and propagated the brain extraction mask to each image. The CRL brain extraction mask included cortical grey matter, white matter, subcortical grey matter structures, cerebrospinal fluid, lateral ventricles and cerebellum. Care was taken to ensure that the mask outline was aligned to coincide with the skull’s inner boundary. It is worth noting that the CRL atlas contains templates from 21 to 37 weeks’ gestation, so template-matching was only possible within the gestational age range that overlapped with our atlas (21 to 31 weeks). For fetuses with scans collected between 14 and 20 weeks’ gestation, the brains were masked using an isotropically scaled version of the earliest CRL atlas brain mask (at 21 weeks’ gestation).

#### Image enhancement and/or hemisphere selection

All images were intensity normalized using histogram matching to a preselected, age-matched reference volume using the imhistmatchn function implemented in MATLAB. Speckle reduction and ridge enhancement (sulci and soft tissue boundaries) were achieved by filtering the images using the contrast-invariant monogenic signal constructed with a multiscale log-Gabor filter. This produced an edge map for each image, which provided a secondary image channel containing enhanced structural information for the atlas construction step (Extended Data Fig. 5b). The processed 3D volumes were classified as clearly capturing the left or right cerebral hemisphere in their distal portion. We reliably ascertained whether the right or left cerebral hemisphere was captured in the image by observing the presentation of the fetal head in the aligned brain volumes. As such, the proximal hemisphere was excluded by cropping the image 10 voxels to the side of the longitudinal fissure, thus removing most of the proximal hemisphere. This step was applied to the images and corresponding edge maps.

### Atlas construction

We created a digital 4D spatiotemporal atlas to characterize fetal brain maturation, encoding the structural variability expected of a healthy population at different gestational ages. We used a diffeomorphic Demons-based approach^59,60^ to estimate the mapping that brought each brain image’s anatomy into spatial correspondence with all other brain images in the population set. As a consequence of the rapid structural evolution of the brain during intrauterine life, we opted for multi-channel groupwise registration^61,62^: a template-free approach to reduce bias introduced by selecting a fixed reference brain image, and the ability to supplement the US intensity images with (several) extra noise-suppressed edge images. That is, no initial template was selected as input to the non-rigid registration; instead, the atlas was completely derived from the set of population images following an iterative optimization procedure. For each gestational age, the algorithm simultaneously generated a deformation map for each population image, and an estimated representation of the average brain template, improving the structural alignment (at the pixel level) with each iteration. The two hemispheres were brought together in the final templates without any anatomically overlapping regions (Fig. 1b and Extended Data Fig. 5c). The ten voxels of the proximal hemisphere that had been retained in the image preparation step were removed to enable the hemispheric templates to be joined along the longitudinal fissure.

**Extended Data Fig. 10:**
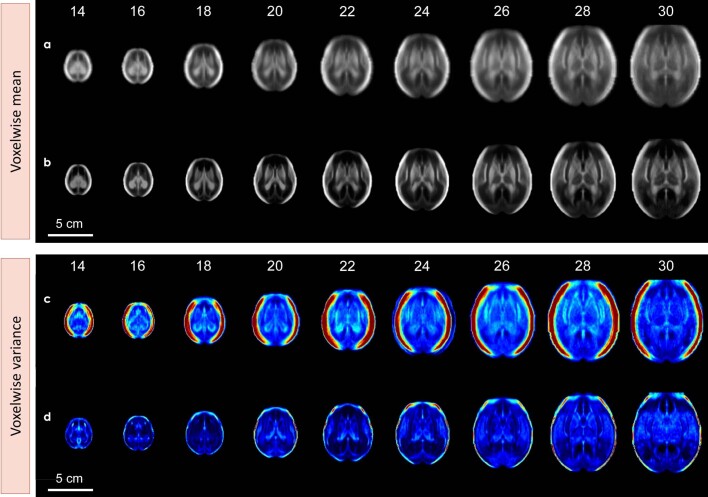
Voxelwise mean and variance maps. before (a, c) and after (b, d) the non-rigid registration step shown for a mid-axial slice (at the level of the thalami) from 14 to 30 weeks’ gestation.

Specifically, for each week and each cerebral hemisphere, the brain template was constructed so that each individual brain was minimally deformed, while the structures were maximally aligned. The algorithm took as input a set ${M}_{{\rm{i}}}^{{\rm{a}}}=\,{\{({{\rm{I}}}_{{\rm{i}}},{{\rm{H}}}_{{\rm{i}}})\,\}}_{\{\,{\rm{i}}=1\}}^{{\rm{N}}}$ comprising *N* pairs of input images and corresponding edge maps, where *a* is the gestational week at which each scan was acquired. Each input image *I*_i_ is a 3D B-mode US scan defined in Euclidean space, and its corresponding structural edge map *H*_i_, was constructed using multiscale feature asymmetry with a log-Gabor filter with kernels of scales $\lambda =\{0.075,0.125,0.175\}$ to enhance fissures while suppressing speckle. The objective was to find a set of non-rigid transformations ${D}_{{\rm{i}}}^{{\rm{a}}}$, each of which mapped the individual images *I*_i_ to a group-representative average image ${\hat{I}}^{{\rm{a}}}$ by simultaneously minimizing the intensity distance across the images at each voxel location (Extended Data Figs. 5 and 10), with additional regularization to enforce a diffeomorphic mapping between the atlas and each individual image^59^ (Extended Data Fig. 3c).

To obtain the age group’s average image ${\hat{I}}^{{\rm{a}}}$, the deformation maps (*D*_i_) were applied to their corresponding images, and the images were combined using a voxel-wise mean (Extended Data Fig. 10). Knowing that the fetal brain evolves over weeks, and that some tissues are transient and may not be consistently observed across the entire gestational age range, we opted to generate a separate atlas for each gestational week. This resulted in 17 brain atlas template maps, spanning 14 to 31 weeks’ gestation (Fig. 1b and Supplementary Video 1).

### Structural atlas labels

Age-specific label maps were generated by manual segmentation of the volumetric atlas images. These were conducted by four authors (A.I.L.N. and F.A.M. for TBV; L.S.H. for subcortical structures; and M.K.W. for CoP), in consultation with histology-based atlases of human fetal brains^63,64^, and were independently verified by two co-authors (A.I.L.N. and W.S.). The atlases were first manually labelled in the axial plane, and iteratively modified in the coronal and sagittal planes by L.S.H. and M.K.W. The following structures were labelled: CoP, CB, ChP and intracranial space (TBV).

The atlas labels were used to train convolutional neural network models to segment the 3D brain mask^6^, subcortical^7^ and cortical^27^ structures automatically. The trained models were then applied to an out-of-sample data set of 3D brain volumes to extract volumetric measures for evaluation of the reliability of the normative patterns of fetal brain growth. Intra-rater variability (between 85 and 91% agreement) was computed for the manual segmentation work related to structural atlas labelling^7^.

The CBV, CoPV and CoPA values reported only cover the period between 18 and 27 weeks’ gestation. Before 18 weeks’ gestation no MRI reference exists and the voxel spacing (0.6 mm), made it difficult to delineate the CB and CoP. After 27 weeks’ gestation, the increased ossification of the skull (for example, the petrous part of the temporal bone for visualizing the CB) reduced image quality.

### Hemispheric asymmetry

We characterized the emergence of local developmental asymmetries between the cerebral hemispheres. For each gestational age, the affinely registered brain images consisting of a clearly visualized right hemisphere were flipped across the midsagittal plane (longitudinal fissure) creating mirror images that matched the left hemisphere maps spatially $({I}_{{\rm{R}}}\to {I}_{{\rm{R}}}^{{\prime} }\approx {I}_{{\rm{L}}})$ (Extended Data Fig. 5d). The analyses were conducted separately for each gestational week to show the timing and regions showing cerebral lateralization.

#### Left and right data distributions

The final selection sample of 899 cases showed a bias towards left visible hemispheres (Extended Data Fig. 1e), which is in keeping with US data, although mostly near term, showing that about two thirds of fetuses are in the left occiput position in utero^65–67^. To verify the effect sizes between scan data sampled from the two hemispheres (for each gestational age), we applied a two-sample Kolmogorov–Smirnov test. Rejection of the null hypothesis confirmed sufficient similarity between the gestational age and brain volumes from each hemisphere. The null hypothesis was rejected for all gestational ages, suggesting that the investigation of asymmetry was valid.

#### Anthropometric asymmetry

The distribution of all volumetric measures for each hemisphere was assessed for normality, conditional on gestational age. Gaussian additive models were fitted to each of the five volumetric measures, separately for each cerebral hemisphere. We then tested whether to reject the null hypothesis of equality (that is, absence of asymmetry) between the left and right hemispheres by computing the Cohen’s *d* estimates for each brain region with the statsmodels Python package (v.0.13.2). The null hypothesis at a nominal 5% level of significance was tested. Significance was found only for ChPV, which produced an *F* statistic of 27.424 on 4 and 847 degrees of freedom, with *P* < 0.001.

#### Spatial asymmetry

To identify spatial patterns of cerebral asymmetry, we performed tensor-based morphometry, which can reveal the local volumetric change (expansion or contraction) between a target and source image. Tensor-based spatial statistics of asymmetry were calculated by applying the logarithm to the diffeomorphic Jacobian determinant maps that resulted from the non-rigid registration step (Atlas construction above). Application of the logarithm encourages the distribution of the deformation fields to be zero-mean and symmetric, which enables ease in interpreting relative tissue growth and/or loss^68^.

Voxel-wise permutation tests were performed on all scans at each gestational age to show which hemisphere had the greater US signal across all scans collected at a given gestational age. We used non-parametric ‘Monte Carlo’ permutation testing as implemented in the FSL RANDOMISE method^69^, and applied threshold-free cluster enhancement to the statistical maps^70^, to enhance the brain areas that showed spatial contiguity. This approach is appropriate when the null distribution is not known a priori, and has been shown to handle noise and spatially correlated signals^70^. We tested for main group effects (left hemisphere, +1, right hemisphere, −1), while including residualized gestational age as a covariate in the general linear model. Five thousand permutations were performed for each contrast, and the regional clusters surviving a conservative family-wise error rate correction threshold of *P* < 0.05 (two-tailed and permutation-based) were deemed as sites of significant asymmetry at the given age. All permutation testing was conducted within the mask of the left cerebral hemisphere (Extended Data Fig. 5d). This tensor-based morphometry approach normalizes for differences in brain volume, and so any detected regions of statistical significance show local morphological, rather than size, differences. In the generated statistical maps (Fig. 5a), positive values shown in the left or right hemisphere indicated either a leftwards (L > R) or rightwards asymmetry (R > L).

We performed secondary analysis of the cerebral subregions to explore the spatial and temporal patterns of asymmetry. Age-matched structural parcellation templates from the CRL atlas were rigidly aligned to our US-based atlas using a shape-preserving similarity transform (scaling, translation, rotation). For each gestational age group, we performed cluster-level analysis to identify the cortical regions showing significant asymmetry. Cluster tables summarize the percentage of cluster-specific voxels contained within each region in the parcellation map (Supplementary Table 8). A surface-based representation of this result was achieved by labelling each cortical region with the percentage of significant voxels within the overlapping cluster. To facilitate visualization of the largest asymmetric regions, only the parcellated regions with at least 10% of voxels having survived permutation testing are shown on the surface. Figure 5d shows the timing of fetal brain lateralization during the second trimester, and the regions of significant difference between the two hemispheres.

### Temporal patterns of maturation

To examine the emergence and evolution of internal brain structures, voxel-wise statistical analyses were performed on pairs of weekly atlases, each separated by 2 weeks, by gathering the scans from the two timepoints (*a* and *b* = *a* + 2) and generating a single groupwise atlas template from all scans (Extended Data Figs. 3 and 4). The atlases were spatially normalized using a global affine transformation to remove size effects. The groupwise registration step yielded voxel-level deformation maps that would map each scan to the central (median) age. That is, the structures in each scan were reconfigured such that the anatomies in the earlier and later gestational timepoints were deformed to the same template, representing the brain at a gestational age between *a* and *b*. Discovery of age-group-specific differences was achieved in two ways. First, we computed the log-Jacobian maps (${J}_{i}^{t}$, which show regions of local structural changes associated with growth or shrinkage) for each deformation field map, and subtracted the mean maps of the two groups:

$${\rm{J}}{\rm{D}}=\frac{1}{{n}_{{\rm{a}}}}\sum _{{n}_{{\rm{a}}}}{J}_{{\rm{i}}}^{{\rm{a}}}-\frac{1}{{{\rm{n}}}_{{\rm{b}}}}\sum _{{{\rm{n}}}_{{\rm{b}}}}{{\rm{J}}}_{{\rm{i}}}^{{\rm{b}}}$$

Positive regions indicate structural expansion from age *a* to *b*, and negative values correspond to regions undergoing shrinkage (Fig. 4a and Extended Data Figs. 3 and 4).

**Extended Data Fig. 11:**
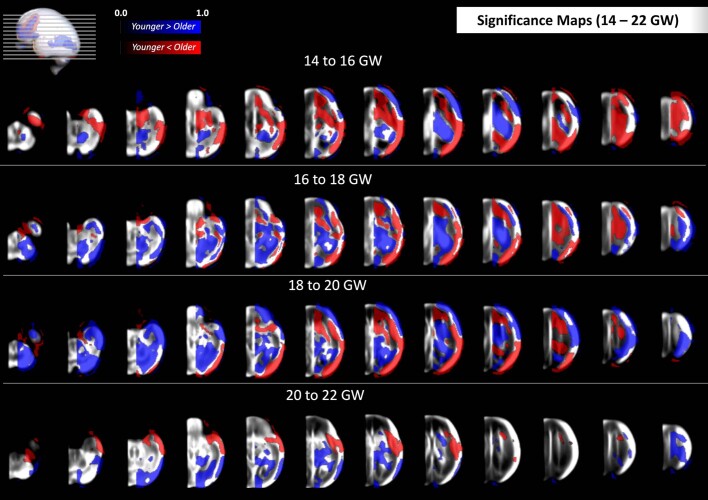
Regions undergoing significant morphological changes from 14 to 22 weeks’ gestation. Statistical maps from our non-parametric analysis of temporal change, overlayed on a set of axial views from bottom (near the cerebellum) to top of the cerebral space. Maps highlight voxel clusters that survived the conservative threshold of $p < 0.05$, shown here for two-week intervals between 14- and 22-weeks’ gestation. Red indicates regions in which the relative (normalised) volume is greater in the younger subgroup ($a$) than in the older subgroup ($a+2$), and vice-versa for blue regions. This figure supports results shown in Fig. 4 of the main text.

**Extended Data Fig. 12:**
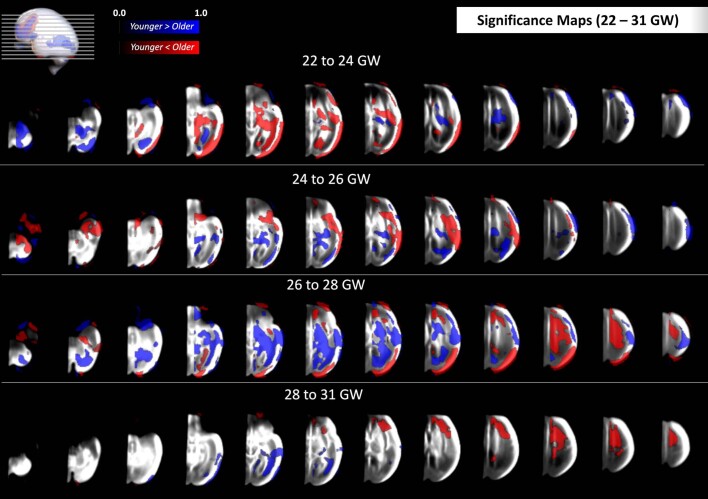
Regions undergoing significant morphological changes from 22 to 31 weeks’ gestation. Statistical maps from our non-parametric analysis of temporal change, overlayed on a set of axial views from bottom (near the cerebellum) to top of the cerebral space. Maps highlight voxel clusters that survived the conservative threshold of $p < 0.05$, shown here for two-week intervals between 22- and 31-weeks’ gestation. Red indicates regions in which the relative (normalised) volume is greater in the younger subgroup ($a$) than in the older subgroup ($a+2$), and vice-versa for blue regions. This figure supports results shown in Fig. 4 of the main text.

Second, we performed voxel-based morphometry with permutation testing implemented in FSL RANDOMISE^69^ with threshold-free cluster enhancement^70^ to highlight regions that were statistically significantly different between the two gestational timepoints. We tested for main group effects by constructing a design matrix with residualized gestational age. Again, 5,000 permutations were performed for each contrast, and only the voxels surviving a conservative threshold of *P* < 0.05 were considered as significantly evolving between the two timepoints (Extended Data Figs. 11 and 12).

### Within-population structural variance

For a given gestational age, each fetus has a spatial map of the amount of voxel-level deformation required for their brain to match that of the population average. By aggregating these deformation fields, we performed voxel-level PCA to determine the breadth of healthy phenotypic structural presentation.

We visually detected a progressive increase in inter-participant variability across the gestational period (Fig. 3e) and for all volumetric measures (Fig. 6), which was confirmed by White’s Lagrangian test for heteroscedasticity^71^.

### Ethics

The INTERGROWTH-21st Project and its ancillary studies were approved by the Oxfordshire Research Ethics Committee ‘C’ (reference no. 08/H0606/139), the research ethics committees of the individual participating institutions, as well as the corresponding regional health authorities where the project was implemented. All mothers provided written informed consent for the use of their clinical data. The sponsors had no role in the study design, data collection, analysis, interpretation of the data, or writing of the paper. The following authors had access to the full raw data set: R.B.G., S.H.K., A.I.L.N., A.P., and J.V. The corresponding author had full access to all the data and final responsibility for submitting the paper.

### Data analysis software statement

Statistical analysis was carried out with the Python statsmodel package (v.0.13.2) and the FSL RANDOMISE tool (https://fsl.fmrib.ox.ac.uk/fsl/fslwiki/Randomise; v.6.0.5). The deep learning models used to perform whole-brain extraction and alignment are available on Github (https://github.com/felipemoser/kelluwen), as is the model used to segment the subcortical structures (https://github.com/lindehesse/FetalSubcortSegm_Code). The atlas was constructed using a script written in MATLAB (v.R2022a), adapted from an implementation of diffeomorphic log-demons image registration (https://www.mathworks.com/matlabcentral/fileexchange/39194-diffeomorphic-log-demons-image-registration). All data analysis scripts were written in Python (v.3.9.6). Plots were generated using the Python seaborn package (v.0.12.1), and cortical surface maps were created using the Python-based ggseg package (v.0.1).

### Reporting summary

Further information on research design is available in the Nature Portfolio Reporting Summary linked to this article.

## Online content

Any methods, additional references, Nature Portfolio reporting summaries, source data, extended data, supplementary information, acknowledgements, peer review information; details of author contributions and competing interests; and statements of data and code availability are available at 10.1038/s41586-023-06630-3.

### Supplementary information

Supplementary Tables Supplementary Tables 1–13.

Reporting Summary

Supplementary Video 1 Evolution of the fetal brain maturation from 14 to 31 weeks’ gestation. The brain volumes have been size normalized to eliminate scaling differences and highlight morphological changes in the brain during this gestational period. Tissue boundaries have been enhanced for clarity.

Supplementary Video 2 Demonstration of methodology for 3D brain image manipulation using a bespoke graphical user interface implemented in MATLAB. Illustrated is the process of rigidly aligning the intracranial region to a common coordinate space, and brain extraction in preparation for atlas construction and extraction of volumetric measures.

## Data Availability

This fetal brain atlas forms part of the INTERGROWTH-21st Project and is publicly available for download (https://intergrowth21.com/research/brain-atlas-project). Owing to the data still being under analysis for the principal and secondary objectives of the study protocol, anonymized image data will be made available on reasonable request for academic use only and within the limitations of the informed consent. Requests must be made to the corresponding author or to the INTERGROWTH-21st Consortium at intergrowth@wrh.ox.ac.uk. Full conditions of access are available in the INTERGROWTH-21st study protocol at https://intergrowth21.com/research/brain-atlas-project. Every request will be reviewed by the INTERGROWTH-21st Consortium Executive Committee with due promptness. After approval, the researcher will need to sign a data access agreement with the INTERGROWTH-21st Consortium.

## References

[1] Garel C, Chantrel E, Elmaleh M, Brisse H, Sebag G (2003). Fetal MRI: normal gestational landmarks for cerebral biometry, gyration and myelination. Childs Nerv. Syst..

[2] Villar J (2014). The likeness of fetal growth and newborn size across non-isolated populations in the INTERGROWTH-21^st^ Project: the Fetal Growth Longitudinal Study and Newborn Cross-Sectional Study. Lancet Diabetes Endocrinol..

[3] de Onis M, Habicht JP (1996). Anthropometric reference data for international use: recommendations from a World Health Organization Expert Committee. Am. J. Clin. Nutr..

[4] Villar J (2018). The satisfactory growth and development at 2 years of age of the INTERGROWTH-21^st^ Fetal Growth Standards cohort support its appropriateness for constructing international standards. Am. J. Obstet. Gynecol..

[5] Villar J (2019). Neurodevelopmental milestones and associated behaviours are similar among healthy children across diverse geographical locations. Nat. Commun..

[6] Moser, F., Huang, R., Papiez, B. W. & Namburete, A. I. L. BEAN: brain extraction and alignment network for 3D fetal neurosonography. *NeuroImage* **258**, 119341 (2022). 10.1016/j.neuroimage.2022.119341 35654376

[7] Hesse LS (2022). Subcortical segmentation of the fetal brain in 3D ultrasound using deep learning. NeuroImage.

[8] Namburete AIL, Xie W, Yaqub M, Zisserman A, Noble JA (2018). Fully-automated alignment of 3D fetal brain ultrasound to a canonical reference space using multi-task learning. Med. Image Anal..

[9] Gholipour A (2017). A normative spatiotemporal MRI atlas of the fetal brain for automatic segmentation and analysis of early brain growth. Sci. Rep..

[10] Chi JG, Dooling EC, Gilles FH (1977). Gyral development of the human brain. Ann. Neurol..

[11] Dubois J (2007). Mapping the early cortical folding process in the preterm newborn brain. Cereb. Cortex.

[12] Samuelsen GB (2003). The changing number of cells in the human fetal forebrain and its subdivisions: a stereological analysis. Cereb. Cortex.

[13] Molnar Z, Kennedy S (2016). Neurodevelopmental disorders: risks of Zika virus during the first trimester of pregnancy. Nat. Rev. Neurol..

[14] Habas PA (2010). A spatiotemporal atlas of MR intensity, tissue probability and shape of the fetal brain with application to segmentation. NeuroImage.

[15] Clouchoux C (2012). Quantitative in vivo MRI measurement of cortical development in the fetus. Brain Struct. Funct..

[16] Yun HJ (2022). Quantification of sulcal emergence timing and its variability in early fetal life: hemispheric asymmetry and sex difference. NeuroImage.

[17] Kolasinski J (2013). Radial and tangential neuronal migration pathways in the human fetal brain: anatomically distinct patterns of diffusion MRI coherence. NeuroImage.

[18] Habas PA (2012). Early folding patterns and asymmetries of the normal human brain detected from in utero MRI. Cereb. Cortex.

[19] Kasprian G (2011). The prenatal origin of hemispheric asymmetry: an in utero neuroimaging study. Cereb. Cortex.

[20] Rajagopalan V (2011). Local tissue growth patterns underlying normal fetal human brain gyrification quantified in utero. J. Neurosci..

[21] Studholme C, Kroenke CD, Dighe M (2020). Motion corrected MRI differentiates male and female human brain growth trajectories from mid-gestation. Nat. Commun..

[22] Bethlehem RAI (2022). Brain charts for the human lifespan. Nature.

[23] Pistorius LR (2010). Grade and symmetry of normal fetal cortical development: a longitudinal two- and three-dimensional ultrasound study. Ultrasound Obstet. Gynecol..

[24] Poon LC (2019). Transvaginal three-dimensional ultrasound assessment of Sylvian fissures at 18-30 weeks’ gestation. Ultrasound Obstet. Gynecol..

[25] Namburete AIL (2015). Learning-based prediction of gestational age from ultrasound images of the fetal brain. Med. Image Anal..

[26] Wyburd, M. K. et al. Assessment of regional cortical development through fissure based gestational age estimation in 3D fetal ultrasound. in *Uncertainty for Safe Utilization of Machine Learning in Medical Imaging, and Perinatal Imaging, Placental and Preterm Image Analysis. UNSURE PIPPI 2021 2021. Lecture Notes in Computer Science* Vol. 12959 (eds Sudre, C. H. et al.) (Springer, 2021).

[27] Wyburd, M. K., Jenkinson, M. & Namburete, A. I. L. Cortical plate segmentation using CNNs in 3D fetal ultrasound. in *Medical Image Understanding and Analysis. MIUA 2020. Communications in Computer and Information Science* Vol. 1248 (eds Papież, B. et al.) (Springer, 2020).

[28] Villar J (2021). Fetal cranial growth trajectories are associated with growth and neurodevelopment at 2 years of age: INTERBIO-21^st^ Fetal Study. Nat. Med..

[29] Papageorghiou AT (2014). International standards for fetal growth based on serial ultrasound measurements: the Fetal Growth Longitudinal Study of the INTERGROWTH-21^st^ Project. Lancet.

[30] Rodriguez-Sibaja, M. J. et al. Fetal cerebellar growth and Sylvian fissure maturation: international standards from the Fetal Growth Longitudinal Study of the INTERGROWTH-21^st^ Project. *Ultrasound Obstet. Gynecol.*10.1002/uog.22017 (2020). 10.1002/uog.22017 32196791

[31] Napolitano R (2020). International standards for fetal brain structures based on serial ultrasound measurements from Fetal Growth Longitudinal Study of INTERGROWTH-21^st^ Project. Ultrasound Obstet. Gynecol..

[32] Kostović I, Judaš M, Radoš M, Hrabač P (2002). Laminar organization of the human fetal cerebrum revealed by histochemical markers and magnetic resonance imaging. Cereb. Cortex.

[33] Khan S (2019). Fetal brain growth portrayed by a spatiotemporal diffusion tensor MRI atlas computed from in utero images. NeuroImage.

[34] Wilson, S. et al. Development of human white matter pathways in utero over the second and third trimester. *Proc. Natl Acad. Sci. USA*10.1073/pnas.2023598118 (2021). 10.1073/pnas.2023598118 PMC8157930 33972435

[35] de Onis M, Garza C, Onyango AW, Martorell R (2006). WHO Child Growth Standards. Acta Paediatr. Suppl..

[36] Desikan RS (2006). An automated labeling system for subdividing the human cerebral cortex on MRI scans into gyral based regions of interest. NeuroImage.

[37] Liu Z (2020). Anatomical and diffusion MRI brain atlases of the fetal rhesus macaque brain at 85, 110 and 135 days gestation. NeuroImage.

[38] Quarello E, Stirnemann J, Ville Y, Guibaud L (2008). Assessment of fetal Sylvian fissure operculization between 22 and 32 weeks: a subjective approach. Ultrasound Obstet. Gynecol..

[39] Broere-Brown ZA (2016). Sex-specific differences in fetal and infant growth patterns: a prospective population-based cohort study. Biol. Sex Diff..

[40] Fernandes M (2020). INTERGROWTH-21st Project international INTER-NDA standards for child development at 2 years of age: an international prospective population-based study. BMJ Open.

[41] Toga AW, Thompson PM (2003). Mapping brain asymmetry. Nat. Rev. Neurosci..

[42] Kong XZ (2018). Mapping cortical brain asymmetry in 17,141 healthy individuals worldwide via the ENIGMA Consortium. Proc. Natl Acad. Sci. USA.

[43] Mallela AN, Deng H, Brisbin AK, Bush A, Goldschmidt E (2020). Sylvian fissure development is linked to differential genetic expression in the pre-folded brain. Sci. Rep..

[44] Bisiacchi P, Cainelli E (2022). Structural and functional brain asymmetries in the early phases of life: a scoping review. Brain Struct. Funct..

[45] Vasung L (2020). Quantitative in vivo MRI assessment of structural asymmetries and sexual dimorphism of transient fetal compartments in the human brain. Cereb. Cortex.

[46] Clouchoux C, Guizard N, Evans AC, du Plessis AJ, Limperopoulos C (2012). Normative fetal brain growth by quantitative in vivo magnetic resonance imaging. Am. J. Obstet. Gynecol..

[47] Schlotz W, Phillips DI (2009). Fetal origins of mental health: evidence and mechanisms. Brain Behav. Immun..

[48] Mowinckel AM, Vidal-Pineiro D (2020). Visualization of brain statistics with R packages ggseg and ggseg3d. Adv. Methods Pract. Psychol. Sci..

[49] Robinson HP, Fleming JE (1975). A critical evaluation of sonar ‘crown-rump length’ measurements. Br. J. Obstet. Gynaecol..

[50] de Onis M, Onyango AW, Van den Broeck J, Chumlea WC, Martorell R (2004). Measurement and standardization protocols for anthropometry used in the construction of a new international growth reference. Food Nutr. Bull..

[51] Victora CG (2008). Maternal and child undernutrition: consequences for adult health and human capital. Lancet.

[52] Fernandes M (2014). The INTERGROWTH-21^st^ Project Neurodevelopment Package: a novel method for the multi-dimensional assessment of neurodevelopment in pre-school age children. PLoS ONE.

[53] Murray E (2018). Evaluation of the INTERGROWTH-21^st^ Neurodevelopment Assessment (INTER-NDA) in 2 year-old children. PLoS ONE.

[54] Koo TK, Li MY (2016). A guideline of selecting and reporting intraclass correlation coefficients for reliability research. J. Chiropr. Med..

[55] Achenbach, T. M. *Manual for the Youth Self-Report and 1991 Profile* (Univ. Vermont Department of Psychiatry, 1991).

[56] Adoh TO, Woodhouse JM, Oduwaiye KA (1992). The Cardiff Test: a new visual acuity test for toddlers and children with intellectual impairment. A preliminary report. Optom. Vis. Sci..

[57] WHO Multicentre Growth Reference Study Group. (2006). WHO Motor Development Study: windows of achievement for six gross motor development milestones. Acta Paediatr. Suppl..

[58] Holmes CJ (1998). Enhancement of MR images using registration for signal averaging. J. Comput. Assist. Tomog..

[59] Vercauteren T, Pennec X, Perchant A, Ayache N (2009). Diffeomorphic demons: efficient non-parametric image registration. NeuroImage.

[60] Papież, B. W., Matuszewski, B. J., Shark, L. K. & Quan, W. in *Mathematical Methodologies in Pattern Recognition and Machine Learning* (eds Latorre Carmona, P. et al.) Springer Proceedings in Mathematics & Statistics, Vol. 30 (Springer, 2013).

[61] Namburete, A. I. L., van Kampen, R., Papageorghiou, A. T. & Papież, B. W. Multi-channel groupwise registration to construct an ultrasound-specific fetal brain atlas. in *Data Driven Treatment Response Assessment and Preterm, Perinatal, and Paediatric Image Analysis. PIPPI DATRA 2018 2018. Lecture Notes in Computer Science* Vol. 11076 (eds Melbourne, A. et al.) (Springer, 2018).

[62] Geng X, Christensen GE, Gu H, Ross TJ, Yang Y (2009). Implicit reference-based group-wise image registration and its application to structural and functional MRI. NeuroImage.

[63] Feess-Higgins, A. & Larroche, J. C. *Development of the Human Fetal Brain Anatomical Atlas* (Institut Natl De LA Sante, 1988).

[64] Bayer, S. A. & Altman, J. *The Human Brain During the Second Trimester* (Taylor & Francis, 2005).

[65] Ahmad, A. et al. Association between fetal position at onset of labor and mode of delivery: a prospective cohort study. *Ultrasound Obstet. Gynecol.* **43**, 176–182 (2014). 10.1002/uog.13189 23929533

[66] Scheer, K. & Nubar, J. Variation of fetal presentation with gestational age. *Am. J. Obstet. Gynecol.* **125**, 269–270 (1976). 10.1016/0002-9378(76)90609-8 1266909

[67] Hughey, M. J. Fetal position during pregnancy. *Am. J. Obstet. Gynecol.* **153**, 885–886 (1985). 10.1016/s0002-9378(85)80276-3 3907357

[68] Leow AD (2007). Statistical properties of Jacobian maps and the realization of unbiased large-deformation nonlinear image registration. IEEE Trans. Med. Imaging.

[69] Winkler AM, Ridgway GR, Webster MA, Smith SM, Nichols TE (2014). Permutation inference for the general linear model. NeuroImage.

[70] Smith SM, Nichols TE (2009). Threshold-free cluster enhancement: addressing problems of smoothing, threshold dependence and localisation in cluster inference. Neuroimage.

[71] White H (1980). A Heteroskedasticity-consistent covariance matrix estimator and a direct test for heteroskedasticity. Econometrica.

