# Supplementary material for: Normative spatiotemporal fetal brain maturation with satisfactory development at 2 years
Source: Nature. 2023 Oct 25;623(7985):106–14. doi: 10.1038/s41586-023-06630-3 (PMC10620088; doi:10.1038/s41586-023-06630-3)
Supplement: Supplementary file 1 — Supplementary Tables 1–13. [file 41586_2023_6630_MOESM1_ESM.docx]

**Supplementary Table 1.** Variance component analysis for total brain volume (TBV, *n=*1059, 14-30 weeks’ gestation), cerebellar volume (CBV, *n=*534, 18-26 weeks’ gestation), choroid plexus volume (ChPV, *n=*851, 14-30 weeks’ gestation), cortical plate volume (CoPV, *n=*534, 18-26 weeks’ gestation), and cortical plate surface area (CoPA, *n=*534, 18-26 weeks’ gestation).

|  | **TBV** | | **CBV** | | **ChPV** | | **CoPV** | | **CoPA** | |
| --- | --- | --- | --- | --- | --- | --- | --- | --- | --- | --- |
|  | *Variance estimate* | *Proportion of variance (%)* | *Variance estimate* | *Proportion of variance (%)* | *Variance estimate* | *Proportion of variance (%)* | *Variance estimate* | *Proportion of variance (%)* | *Variance estimate* | *Proportion of variance (%)* |
| Variance between sites | 1.94 | 1.27 | 1.19 | 1.56 | 1.36 | 1.12 | 6.34 | 7.78 | 4.19 | 5.28 |
| Residual variance | 3.21x10^5^ | 98.72 | 77.2 | 98.44 | 17.7 | 98.88 | 1.18x10^10^ | 92.21 | 6.09x10^9^ | 94.72 |

**Supplementary Table 2.** Structural brain growth comparisons between male and female fetuses expressed as percentage increases in absolute structural volumes ($S_{i}$) from the female to the male brain at 22- and 27-weeks’ gestation.

| Structural measure (S) | $\boldsymbol{S}_{\text{M}}\boldsymbol{-}\boldsymbol{S}_{\text{F}}$ (%) | | P-value | | | | | |
| --- | --- | --- | --- | --- | --- | --- | --- | --- |
|  | @22 GW | @27 GW | GA | (M\|F) | (M\|F) x GA | (M\|F) x (R\|L) | (R\|L) | (R\|L) x GA |
| TBV | + 6.14 | + 5.94 | **<0.001** | **0.049** | 0.999 | 0.146 | 0.053 | **<0.001** |
| ChPV | - 4.49 | - 2.15 | **0.010** | **0.047** | 0.185 | 0.870 | **0.010** | 0.395 |
| CBV | + 5.69 | + 6.48 | **<0.001** | 0.860 | 0.714 | 0.216 | 0.071 | 0.090 |
| CoPV | + 5.13 | + 9.21 | **<0.001** | 0.081 | **0.017** | 0.852 | 0.139 | 0.120 |
| CoPA | + 4.13 | + 6.48 | **<0.001** | 0.247 | 0.052 | 0.835 | 0.464 | 0.571 |

Uncorrected p-values for the difference in the growth models generated for each parameter. Boldface $p<0.05$.

TBV: Total brain volume; ChPV: choroid plexus volume; CBV: cerebellar volume; CoPV: cortical plate volume; CoPA: cortical plate surface area.

(M|F): sex, (R|L): hemisphere, GA: gestational age

**Supplementary Table 3.** Birth measures, and measures and neurodevelopmental outcomes at 2 years of age of infants included in the follow-up to the INTERGROWTH-21^st^ Fetal Growth Longitudinal Study (FGLS), whose fetal brain images were analysed, compared to the total FGLS population.

|  | **Optimal images (Brain atlas)** | | | **Moderately optimal brain images** | | **INTERGROWTH-21^st^**  **total FGLS population** | |
| --- | --- | --- | --- | --- | --- | --- | --- |
|  |  | |  | | |  | |
| **Newborns measured** | n=899 | | n=1294 | | | n=4321 | |
| *Mean ± SD* |  | |  | | |  | |
| Weight (kg) | 3.2 (0.4) | | 3.3 (0.4) | | | 3.3 (0.4) | |
| Length (cm) | 49.2 (1.9) | | 49.6 (1.8) | | | 49.4 (1.9) | |
| Head circumference (cm) | 33.8 (1.2) | | 34.0 (1.2) | | | 33.9 (1.3) | |
|  |  | |  | | |  | |
| **Infants measured at age 2**  *Mean ± SD* | n=671 | | n=929 | | | n=3042 | |
| Weight (z-score) | 0.2 (1.1) | | 0.3 (1.0) | | | 0.2 (1.1) | |
| Height (z-score) | 0.1 (1.1) | | 0.2 (1.0) | | | 0.0 (1.1) | |
| Head circumference (z-score) | 0.1 (1.1) | | 0.1 (1.1) | | | 0.0 (1.1) | |
|  |  | |  | | |  | |
| **Infants assessed with the**  **INTER-NDA at age 2** | n=246 | | n=346 | | | n=1181 | |
| *Mean (± SD) and*  **Mean (normative range)* |  |  | | |  | |  |
| Cognitive^1^ | 71.7 (21.7) | | 73.5 (20.7) | | | 71.8 (38.5-99.6)* | |
| Language^1^ | 61.9 (28.4) | | 66.7 (26.7) | | | 63.5 (17.8-100)* | |
| Fine motor^1^ | 78.2 (29.1) | | 81.6 (25.6) | | | 78.5 (25.7-100)* | |
| Gross motor^1^ | 78.7 (22.6) | | 80.5 (20.8) | | | 79.6 (51.7-100)* | |
|  |  | |  | | |  | |
| *Emotional affect*, *Mean (IQR)*  *and *Mean (normative range)* |  | |  | | |  | |
| Positive affect^1^ | 90 (70-100) | | 90 (70-100) | | | 90 (51.4-100)* | |
| Negative affect^2^ | 25 (0-25) | | 25 (0-25) | | | 25 (0.0-76.5)* | |
|  |  | |  | | |  | |
| *Vision*, *Median (IQR)* |  | |  | | |  | |
| Visual Acuity (LogMAR) | 0.1 (0.1-0.2) | | 0.2 (0.1-0.2) | | | 0.2 (0.1-0.2) | |
| Contrast sensitivity (%) | 1.5 (1.0-1.5) | | 1.5 (1.0-2.0) | | | 1.5 (1.0-2.0) | |

^1^ For these domains, higher scores reflect better outcome; ^2^ For negative behaviour, lower scores reflect better outcomes

IQR: inter-quartile range; logMAR: Logarithm of the Minimum Angle of Resolution

**Supplementary Table 4.** Maternal and perinatal characteristics of pregnancies in the INTERGROWTH-21^st^ Fetal Growth Longitudinal Study (FGLS) included in the analysis of optimal fetal brain images compared to the total FGLS population.

| **Characteristics** | **Optimal images** | **FGLS total population** | **P value^¥^** |
| --- | --- | --- | --- |
|  | **n=899** | **n=4321** |  |
| Maternal age (years) | 28.2 ± 3.8 | 28.4 ± 3.9 | 0.240 |
| Maternal height (cm) | 161.6 ± 5.5 | 162.2 ± 5.8 | 0.004 |
| Maternal weight (Kg) | 57.4 ± 7.9 | 61.3 ± 9.1 | 0.000 |
| Paternal height (cm) | 173.9 ± 7.4 | 174.4 ± 7.3 | 0.099 |
| Body mass index, Kg/m2 | 22.0 ± 2.6 | 23.3 ± 3.0 | 0.000 |
| Gestational age at first visit (weeks) | 11.7 ± 1.4 | 11.7 ± 1.4 | 0.947 |
| Time in formal education (years) | 14.8 ± 3.0 | 15.0 ± 2.8 | 0.062 |
| Haemoglobin level at <15 weeks’ gestation (g/L) | 124.2 ± 11.3 | 124.7 ± 11.1 | 0.273 |
| Nulliparous | 564 (62.7%) | 2955 (68.4%) | 0.001 |
| Pre-eclampsia | 3 (0.3%) | 31 (0.7%) | 0.192 |
| Pyelonephritis | 3 (0.3%) | 16 (0.4%) | 0.868 |
| Any sexually transmitted infection | 0 (0.0%) | 3 (0.1%) | 0.429 |
| Spontaneous initiation of labour | 642 (71.4%) | 2869 (66.4%) | 0.004 |
| PPROM (<37 weeks’ gestation) | 10 (1.1%) | 82 (1.9%) | 0.103 |
| Caesarean section | 263 (29.3%) | 1541 (35.7%) | 0.000 |
| NICU admission (>1 day) | 35 (3.9%) | 240 (5.6%) | 0.043 |
| Term low birth weight (<2500g) | 30 (3.4%) | 122 (3.0%) | 0.444 |
| Neonatal mortality | 1 (0.1%) | 7 (0.2%) | 0.723 |
| Neonatal male sex | 448 (49.8%) | 2150 (49.8%) | 0.967 |
| Exclusive breastfeeding at discharge | 789 (88.0%) | 3790 (88.0%) | 0.962 |
| Mother admitted to intensive care unit | 2 (0.2%) | 17 (0.4%) | 0.438 |

Data given as mean ± SD or n (%). Data missing for <1% of sample for all variables, unless indicated otherwise. *Data missing for 45% of sample. †Data missing for 32% of sample.

NICU, neonatal intensive care unit; PPROM, preterm prelabour rupture of membranes.

¥ P value of comparison between the two samples. T-test for continuous variables and two-sample test for equality of proportions.

**Supplementary Table 5.** Morbidity at 2 years of age of infants included in the follow-up to the INTERGROWTH-21^st^ Fetal Growth Longitudinal Study (FGLS), whose fetal brain images were analysed, compared to the total FGLS population.

| **Medical condition** | **Optimal images** | **FGLS total population** | **P value^¥^** |
| --- | --- | --- | --- |
|  | **n=675** | **n=3041** |  |
| Hospitalised at least once | 68 (10.1%) | 272 (8.9%) | 0.357 |
| Total no. of days hospitalised | 2 (1-4)ⱡ | 3(1-5)ⱡ | 0.220 |
| Any prescription made by healthcare professional | 465 (68.9%) | 2018 (66.4%) | 0.207 |
| Antibiotics (≤3 regimens) | 119 (17.6%) | 481 (15.8%) | 0.247 |
| Iron/folic acid/vitamin B12/other vitamins | 97 (14.4%) | 431 (14.2%) | 0.894 |
| Up-to-date with local vaccination policies | 646 (95.7%) | 2904 (95.5%) | 0.840 |
| Otitis media/pneumonia/bronchiolitis | 71 (10.5%) | 294 (9.7%) | 0.502 |
| Parasitosis/diarrhoea/vomiting | 24 (3.6%) | 139 (4.6%) | 0.243 |
| Seizures/cerebral palsy/neurological disorders | 2 (0.3%) | 9 (0.3%) | 0.999 |
| Exanthema/skin disease | 86 (12.7%) | 399 (13.1%) | 0.791 |
| UTI/pyelonephritis | 1 (0.1%) | 10 (0.3%) | 0.434 |
| Fever ≥3 d (≥3 episodes) | 57 (8.4%) | 309 (10.2%) | 0.176 |
| Malaria | 0 (0.0%) | 12 (0.4%) | 0.102 |
| Meningitis | 0 (0.0%) | 1 (0.0%) | 0.638 |
| Other infections that required antibiotics | 17 (2.5%) | 79 (2.6%) | 0.906 |
| Hearing problems | 0 (0.0%) | 3 (0.1%) | 0.414 |
| Asthma | 8 (1.2%) | 42 (1.4%) | 0.689 |
| Cardiovascular problems | 3 (0.4%) | 7 (0.2%) | 0.331 |
| Blindness | 0 (0.0%) | 4 (0.1%) | 0.346 |
| Gastroesophageal reflux | 3 (0.4%) | 9 (0.3%) | 0.538 |
| Any haemolytic condition | 6 (0.9%) | 22 (0.7%) | 0.653 |
| Any malignancy | 2 (0.3%) | 6 (0.2%) | 0.616 |
| Cow’s milk protein allergy | 4 (0.6%) | 21 (0.7%) | 0.778 |
| Food allergies | 10 (1.5%) | 52 (1.7%) | 0.675 |
| Injury trauma | 34 (5.0%) | 130 (4.3%) | 0.383 |
| Any condition that required surgery | 8 (1.2%) | 34 (1.1%) | 0.881 |

UTI, urinary tract infection.

ⱡData are given as median (interquartile range).

¥ P value of comparison between the two samples. T-test for continuous variables and two-sample test for equality of proportions.

**Supplementary Table 6.** Image inclusion criteria used for data selection for atlas construction.

| **Overall 3D image** | **Midsagittal Plane** | **Transthalamic plane** | **Transcerebellar plane** |
| --- | --- | --- | --- |
| Absence of maternal or fetal motion artifacts | Cavum septi pellucidi visible | Symmetrical plane | Cerebellum visible |
| Head fully contained within the image | Eye sockets not visible | Head occupying at least 30% of image | Cerebellar vermis visible |
| Minimal acoustic shadows in the distal hemisphere | Facial profile clearly visible (<24 weeks’ gestation) | Cavum septi pellucidi visible | Cisterna magna visible |
| High contrast between fluid-filled (cavum septi pellucidi, ventricles) and dense (e.g. skull, choroid plexus) regions | Choroid plexus not visible (<18 weeks) | Choroid plexus visible within ventricular cavity |  |
|  |  | Thalami visible |  |
|  |  | Posterior ventricle cavity visible |  |
|  |  | Sylvian fissure visible |  |

**Supplementary Table 7.** Number of scans (per week) included in the atlas, summarised by percentage of left versus right hemispheres, female/male subjects.

| **Gestational week** | **Number of scans** | **Number of scans capturing left hemisphere (%)** | **Newborn sex (% female)** |
| --- | --- | --- | --- |
| 14 | 30 | 18 (60.0%) | 33.30% |
| 15 | 52 | 26 (50.0%) | 42.30% |
| 16 | 83 | 37 (44.6%) | 50.60% |
| 17 | 64 | 35 (54.7%) | 45.30% |
| 18 | 87 | 47 (54.0%) | 44.80% |
| 19 | 58 | 33 (56.9%) | 60.30% |
| 20 | 85 | 56 (65.9%) | 52.90% |
| 21 | 78 | 51 (65.4%) | 50.00% |
| 22 | 61 | 42 (68.9%) | 52.50% |
| 23 | 86 | 60 (69.8%) | 47.70% |
| 24 | 58 | 37 (63.8%) | 50.00% |
| 25 | 51 | 41 (80.4%) | 56.90% |
| 26 | 77 | 49 (63.9%) | 55.80% |
| 27 | 58 | 42 (72.4%) | 60.30% |
| 28 | 69 | 51 (73.9%) | 55.10% |
| 29 | 29 | 18 (62.1%) | 55.20% |
| 30 | 33 | 25 (75.8%) | 63.60% |
| **Total** | **1059** | **668 (63.1%)** | **51.20%** |

**Supplementary Table 8.** Cluster table summarising the percentage of asymmetric voxels contained within each region in the Desikan-Killiany parcellation map. Asymmetry was determined using the FSL RANDOMISE method for threshold-free cluster enhancement, with voxels surviving a conservative threshold of *p<0.05* (two-tailed and permutation-tested).

| **RegionID** | | **Gestational age, in weeks** | | | | | |
| --- | --- | --- | --- | --- | --- | --- | --- |
|  |  | **21** | **22** | **23** | **24** | **25** | **26** |
| **Temporal** | Banks STS | 0 | 0 | 0.1 | 0 | 0 | 0 |
|  | Entorhinal | 0 | 0 | 0 | 0 | 0 | 0 |
|  | Fusiform | 0 | 0 | 0 | 0 | 0 | 0 |
|  | Inferior temporal | 0 | 0 | 0 | 0 | 0 | 0 |
|  | Middle temporal | 0 | 0 | 0.1 | 0 | 0 | 0 |
|  | Parahippocampal | 0 | 0 | 0 | 0 | 0 | 0 |
|  | Superior temporal | 0 | 0 | 2.2 | 0 | 0 | 0 |
|  | Temporal pole | 0 | 0 | 0 | 0 | 0 | 0 |
|  | Transverse temporal | 0 | 0 | 14.1 | 0 | 0 | 0 |
|  | Insula | 0 | 0 | 0 | 0 | 0 | 0 |
| **Parietal** | Inferior parietal | 0 | 0 | 0.3 | 3.4 | 0 | 0 |
|  | Paracentral | 0 | 0 | 0 | 12.9 | 0 | 0 |
|  | Postcentral | 0 | 0 | 0 | 0.5 | 0 | 0 |
|  | Precuneus | 0 | 0 | 0 | 0.7 | 0 | 0 |
|  | Superior parietal | 0 | 0 | 0 | 29.4 | 0 | 0 |
|  | Supramarginal | 0 | 0 | 7.9 | 0 | 0 | 0 |
| **Frontal** | Caudal middle frontal | 23.9 | 0 | 0 | 0 | 0 | 0 |
|  | Lateral orbitofrontal | 51.7 | 0 | 0 | 0 | 0 | 0 |
|  | Medial orbitofrontal | 2.1 | 0 | 0 | 0 | 0 | 0 |
|  | Pars opercularis | 0 | 0 | 0 | 0 | 0 | 0 |
|  | Pars orbitalis | 6.2 | 0 | 0 | 0 | 0 | 0 |
|  | Pars triangularis | 12.5 | 0 | 0 | 0 | 0 | 0 |
|  | Rostral middle frontal | 23.9 | 0 | 0 | 0 | 0 | 0 |
|  | Superior frontal | 4.4 | 0 | 0 | 0 | 0 | 0 |
|  | Frontal pole | 2.1 | 0 | 0 | 0 | 0 | 0 |
|  | Precentral | 0 | 0 | 0 | 0 | 0 | 0 |
| **Occipital** | Cuneus | 0 | 0 | 0 | 0 | 0 | 0 |
|  | Lateral occipital | 0 | 0 | 0 | 0 | 0 | 0 |
|  | Lingual | 0 | 0 | 0 | 0 | 0 | 0 |
|  | Pericalcarine | 0 | 0 | 0 | 0 | 0 | 0 |
| **Cingulate** | Caudal anterior cingulate | 0 | 0 | 0 | 0.4 | 0 | 0 |
|  | Isthmus cingulate | 0 | 0 | 0 | 0.9 | 0 | 0 |
|  | Posterior cingulate | 0 | 0 | 0 | 3.5 | 0 | 0 |
|  | Rostral anterior cingulate | 0 | 0 | 0 | 0.4 | 0 | 0 |

**Supplementary Table 9.** Standardised site discrepancy (SSD) for total brain volume (TBV, n=1059 three-dimensional volumes). SSD calculated as: (mean of site's measurements – mean of all sites' measurements at each gestational age interval)/standard deviation of all sites' measurements at each gestational age interval.

| All sites and individual site sample sizes, means and standard deviations (SD) for TBV (mm^3^) | | | | | |
| --- | --- | --- | --- | --- | --- |
| GA (weeks) | Country | Sample | TBV (mm^3^) | TBV (mm^3^) | Standardised |
|  |  | total | Adj. Mean | Adj. SD | Effect |
| 14 to 18 | Brazil | 33 | 23.03 | 3.45 | 0.21 |
|  | China | 57 | 21.83 | 3.74 | -0.13 |
|  | India | 38 | 21.92 | 3.76 | -0.10 |
|  | Kenya | 35 | 22.58 | 3.53 | 0.08 |
|  | Oman | 63 | 22.54 | 3.09 | 0.08 |
|  | UK | 49 | 21.60 | 3.52 | -0.20 |
|  | USA | 12 | 22.90 | 3.80 | 0.16 |
|  | Italy | 30 | 23.33 | 3.21 | 0.32 |
|  | **All sites** | **317** | **22.30** | **3.48** | **0.00** |
|  |  |  |  |  |  |
| 19 to 23 | Brazil | 31 | 66.74 | 8.66 | 0.19 |
|  | China | 70 | 62.61 | 8.48 | -0.30 |
|  | India | 41 | 63.74 | 9.22 | -0.15 |
|  | Kenya | 57 | 67.58 | 8.72 | 0.28 |
|  | Oman | 80 | 65.41 | 7.51 | 0.04 |
|  | UK | 38 | 63.59 | 8.02 | -0.19 |
|  | USA | 14 | 67.21 | 9.86 | 0.21 |
|  | Italy | 37 | 68.56 | 7.10 | 0.48 |
|  | **All sites** | **368** | **65.13** | **8.34** | **0.00** |
|  |  |  |  |  |  |
| 24 to 28 | Brazil | 15 | 141.90 | 16.72 | 0.18 |
|  | China | 61 | 139.72 | 18.30 | 0.04 |
|  | India | 48 | 134.36 | 17.13 | -0.27 |
|  | Kenya | 24 | 141.92 | 19.16 | 0.16 |
|  | Oman | 49 | 135.76 | 12.88 | -0.25 |
|  | UK | 57 | 139.27 | 16.08 | 0.02 |
|  | USA | 10 | 146.96 | 17.90 | 0.45 |
|  | Italy | 48 | 143.40 | 15.72 | 0.29 |
|  | **All sites** | **312** | **138.91** | **16.27** | **0.00** |
|  |  |  |  |  |  |
| 29 to 33 | Brazil | 7 | 207.24 | 22.27 | 0.09 |
|  | China | 5 | 200.43 | 18.45 | -0.26 |
|  | India | 9 | 202.87 | 28.82 | -0.08 |
|  | Kenya | 8 | 207.13 | 22.40 | 0.08 |
|  | Oman | 24 | 202.22 | 20.37 | -0.15 |
|  | UK | 7 | 208.17 | 19.13 | 0.15 |
|  | USA | 0 | 216.11 | 21.85 | 0.50 |
|  | Italy | 2 | 215.52 | 18.66 | 0.55 |
|  | **All sites** | **62** | **324.25** | **11.83** | **0.00** |
|  |  |  |  |  |  |
|  |  |  |  |  |  |
| **Standardised site effects:** Refers to the differences between the observed site means and the | | | | | |
| corresponding predicted site means divided by the predicted pooled SD | | | | | |
| **Adj. Mean:** Refers to the equivalent mean at the median gestational age category | | | | | |
| **Adj. SD:** Refers to the equivalent SD at the median gestational age category | | | | | |

**Supplementary Table 10.** Standardised site discrepancy (SSD) for cerebellar volume (CBV, n=534 three-dimensional volumes). SSD calculated as: (mean of site's measurements – mean of all sites' measurements at each gestational age interval)/standard deviation of all sites' measurements at each gestational age interval.

| All sites and individual site sample sizes, means and standard deviations (SD) for CBV (mm^3^) | | | | | |
| --- | --- | --- | --- | --- | --- |
| GA (weeks) | Country | Sample | CBV (mm^3^) | CBV (mm^3^) | Standardised |
|  |  | total | Adj. Mean | Adj. SD | Effect |
| 18 to 23 | Brazil | 25 | 1.65 | 0.43 | -0.28 |
|  | China | 82 | 1.71 | 0.43 | -0.13 |
|  | India | 42 | 1.82 | 0.38 | 0.13 |
|  | Kenya | 62 | 1.95 | 0.40 | 0.45 |
|  | Oman | 77 | 1.83 | 0.31 | 0.18 |
|  | UK | 46 | 1.67 | 0.37 | -0.27 |
|  | USA | 16 | 1.78 | 0.39 | 0.03 |
|  | Italy | 28 | 1.81 | 0.48 | 0.09 |
|  | **All sites** | **378** | **1.77** | **0.40** | **0.00** |
|  |  |  |  |  |  |
| 24 to 28 | Brazil | 9 | 3.49 | 0.82 | -0.54 |
|  | China | 8 | 4.10 | 0.54 | 0.31 |
|  | India | 28 | 3.91 | 0.74 | -0.02 |
|  | Kenya | 14 | 4.08 | 0.80 | 0.18 |
|  | Oman | 37 | 4.01 | 0.66 | 0.13 |
|  | UK | 22 | 3.82 | 0.70 | -0.15 |
|  | USA | 7 | 4.12 | 0.60 | 0.32 |
|  | Italy | 31 | 4.01 | 0.92 | 0.09 |
|  | **All sites** | **156** | **3.93** | **0.74** | **0.00** |
|  |  |  |  |  |  |
|  |  |  |  |  |  |
| **Standardised site effects:** Refers to the differences between the observed site means and the | | | | | |
| corresponding predicted site means divided by the predicted pooled SD | | | | | |
| **Adj. Mean:** Refers to the equivalent mean at the median gestational age category | | | | | |
| **Adj. SD:** Refers to the equivalent SD at the median gestational age category | | | | | |

**Supplementary Table 11.** Standardised site discrepancy (SSD) for choroid plexus volume (ChPV, n=635 three-dimensional volumes). SSD calculated as: (mean of site's measurements – mean of all sites' measurements at each gestational age interval)/standard deviation of all sites' measurements at each gestational age interval.

| All sites and individual site sample sizes, means and standard deviations (SD) for ChPV (mm^3^) | | | | | |
| --- | --- | --- | --- | --- | --- |
| GA (weeks) | Country | Sample | ChPV (mm^3^) | ChPV (mm^3^) | Standardised |
|  |  | total | Adj. Mean | Adj. SD | Effect |
| 18 to 23 | Brazil | 25 | 0.66 | 0.18 | 0.02 |
|  | China | 82 | 0.65 | 0.14 | -0.09 |
|  | India | 42 | 0.64 | 0.15 | -0.12 |
|  | Kenya | 62 | 0.68 | 0.15 | 0.13 |
|  | Oman | 77 | 0.66 | 0.15 | -0.02 |
|  | UK | 46 | 0.66 | 0.16 | -0.04 |
|  | USA | 16 | 0.74 | 0.11 | 0.71 |
|  | Italy | 28 | 0.68 | 0.18 | 0.10 |
|  | **All sites** | **378** | **0.66** | **0.16** | **0.00** |
|  |  |  |  |  |  |
| 24 to 28 | Brazil | 11 | 0.73 | 0.18 | -0.13 |
|  | China | 50 | 0.75 | 0.06 | 0.02 |
|  | India | 37 | 0.73 | 0.15 | -0.12 |
|  | Kenya | 20 | 0.73 | 0.18 | -0.12 |
|  | Oman | 39 | 0.78 | 0.17 | 0.18 |
|  | UK | 47 | 0.74 | 0.20 | -0.04 |
|  | USA | 10 | 0.78 | 0.18 | 0.19 |
|  | Italy | 43 | 0.72 | 0.22 | -0.15 |
|  | **All sites** | **257** | **0.75** | **0.18** | **0.00** |
|  |  |  |  |  |  |
|  |  |  |  |  |  |
| **Standardised site effects:** Refers to the differences between the observed site means and the | | | | | |
| corresponding predicted site means divided by the predicted pooled SD | | | | | |
| **Adj. Mean:** Refers to the equivalent mean at the median gestational age category | | | | | |
| **Adj. SD:** Refers to the equivalent SD at the median gestational age category | | | | | |

**Supplementary Table 12.** Standardised site discrepancy (SSD) for cortical plate volume (CoPV, n=534 three-dimensional volumes). SSD calculated as: (mean of site's measurements – mean of all sites' measurements at each gestational age interval)/standard deviation of all sites' measurements at each gestational age interval.

| All sites and individual site sample sizes, means and standard deviations (SD) for CoPV (mm^3^) | | | | | |
| --- | --- | --- | --- | --- | --- |
| GA (weeks) | Country | Sample | CoPV (mm^3^) | CoPV (mm^3^) | Standardised |
|  |  | total | Adj. Mean | Adj. SD | Effect |
| 18 to 23 | Brazil | 25 | 10.90 | 1.44 | 0.13 |
|  | China | 82 | 10.38 | 1.27 | -0.26 |
|  | India | 42 | 10.58 | 1.45 | -0.08 |
|  | Kenya | 62 | 11.38 | 1.60 | 0.42 |
|  | Oman | 77 | 10.71 | 1.31 | 0.00 |
|  | UK | 46 | 10.35 | 1.25 | -0.28 |
|  | USA | 16 | 10.98 | 1.55 | 0.18 |
|  | Italy | 28 | 11.41 | 1.57 | 0.45 |
|  | **All sites** | **378** | **10.71** | **1.41** | **0.00** |
|  |  |  |  |  |  |
| 24 to 28 | Brazil | 9 | 22.89 | 2.51 | -0.03 |
|  | China | 8 | 22.08 | 1.74 | -0.51 |
|  | India | 28 | 22.69 | 2.63 | -0.11 |
|  | Kenya | 14 | 23.54 | 2.44 | 0.23 |
|  | Oman | 37 | 22.66 | 2.16 | -0.14 |
|  | UK | 22 | 22.91 | 2.02 | -0.03 |
|  | USA | 7 | 23.41 | 2.13 | 0.21 |
|  | Italy | 31 | 23.94 | 2.03 | 0.48 |
|  | **All sites** | **156** | **22.97** | **2.25** | **0.00** |
|  |  |  |  |  |  |
|  |  |  |  |  |  |
| **Standardised site effects:** Refers to the differences between the observed site means and the | | | | | |
| corresponding predicted site means divided by the predicted pooled SD | | | | | |
| **Adj. Mean:** Refers to the equivalent mean at the median gestational age category | | | | | |
| **Adj. SD:** Refers to the equivalent SD at the median gestational age category | | | | | |

**Supplementary Table 13.** Standardised site discrepancy (SSD) for cortical plate surface area (CoPA, n=534 three-dimensional volumes). SSD calculated as: (mean of site's measurements – mean of all sites' measurements at each gestational age interval)/standard deviation of all sites' measurements at each gestational age interval.

| All sites and individual site sample sizes, means and standard deviations (SD) for CoPA (mm^2^) | | | | | |
| --- | --- | --- | --- | --- | --- |
| GA (weeks) | Country | Sample | CoPA (mm^2^) | CoPA (mm^2^) | Standardised |
|  |  | total | Adj. Mean | Adj. SD | Effect |
| 18 to 23 | Brazil | 25 | 89.05 | 8.31 | 0.01 |
|  | China | 82 | 87.45 | 7.85 | -0.19 |
|  | India | 42 | 88.05 | 9.27 | -0.10 |
|  | Kenya | 62 | 93.40 | 9.36 | 0.48 |
|  | Oman | 77 | 89.04 | 7.70 | 0.01 |
|  | UK | 46 | 86.46 | 7.06 | -0.35 |
|  | USA | 16 | 91.33 | 9.82 | 0.24 |
|  | Italy | 28 | 93.06 | 8.49 | 0.49 |
|  | **All sites** | **378** | **88.93** | **8.36** | **0.00** |
|  |  |  |  |  |  |
| 24 to 28 | Brazil | 9 | 158.23 | 11.17 | -0.27 |
|  | China | 8 | 159.13 | 10.51 | -0.20 |
|  | India | 28 | 159.47 | 13.10 | -0.13 |
|  | Kenya | 14 | 162.95 | 13.78 | 0.13 |
|  | Oman | 37 | 161.29 | 11.47 | 0.01 |
|  | UK | 22 | 160.80 | 10.49 | -0.04 |
|  | USA | 7 | 165.21 | 10.91 | 0.37 |
|  | Italy | 31 | 163.40 | 9.98 | 0.22 |
|  | **All sites** | **156** | **161.21** | **11.48** | **0.00** |
|  |  |  |  |  |  |
|  |  |  |  |  |  |
| **Standardised site effects:** Refers to the differences between the observed site means and the | | | | | |
| corresponding predicted site means divided by the predicted pooled SD | | | | | |
| **Adj. Mean:** Refers to the equivalent mean at the median gestational age category | | | | | |
| **Adj. SD:** Refers to the equivalent SD at the median gestational age category | | | | | |
