# Supplementary material for: Normative spatiotemporal fetal brain maturation with satisfactory development at 2 years
Source: Nature. 2023 Oct 25;623(7985):106–14. doi: 10.1038/s41586-023-06630-3 (PMC10620088; doi:10.1038/s41586-023-06630-3)
Supplement: Supplementary file 2 — Reporting Summary [file 41586_2023_6630_MOESM2_ESM.pdf]

Corresponding author(s): Ana I.L. Namburete
Last updated by author(s): Jun 5, 2024

Reporting Summary

Nature Portfolio wishes to improve the reproducibility of the work that we publish. This form provides structure for consistency and transparency in reporting. For further information on Nature Portfolio policies, see our Editorial Policies and the Editorial Policy Checklist.

Statistics

For all statistical analyses, confirm that the following items are present in the figure legend, table legend, main text, or Methods section.

n/a	Confirmed	
☐	☒	The exact sample size (*n*) for each experimental group/condition, given as a discrete number and unit of measurement
☐	☒	A statement on whether measurements were taken from distinct samples or whether the same sample was measured repeatedly
☐	☒	The statistical test(s) used AND whether they are one- or two-sided<br>*Only common tests should be described solely by name; describe more complex techniques in the Methods section.*
☐	☒	A description of all covariates tested
☐	☒	A description of any assumptions or corrections, such as tests of normality and adjustment for multiple comparisons
☐	☒	A full description of the statistical parameters including central tendency (e.g. means) or other basic estimates (e.g. regression coefficient) AND variation (e.g. standard deviation) or associated estimates of uncertainty (e.g. confidence intervals)
☐	☒	For null hypothesis testing, the test statistic (e.g. *F*, *t*, *r*) with confidence intervals, effect sizes, degrees of freedom and *P* value noted<br>*Give P values as exact values whenever suitable.*
☒	☐	For Bayesian analysis, information on the choice of priors and Markov chain Monte Carlo settings
☒	☐	For hierarchical and complex designs, identification of the appropriate level for tests and full reporting of outcomes
☒	☐	Estimates of effect sizes (e.g. Cohen's *d*, Pearson's *r*), indicating how they were calculated

Our web collection on statistics for biologists contains articles on many of the points above.

Software and code

Policy information about availability of computer code

Data collection	No specialised software was used for data collection.
Data analysis	Statistical analysis was performed with the Python statsmodel package (version 0.13.2) and the FSL RANDOMISE tool (https://fsl.fmrib.ox.ac.uk/fsl/fslwiki/Randomise; version 6.0.5). The deep learning models used to perform whole-brain extraction and alignment are available on Github (https://github.com/felipemoser/kelluwen), as is the model used to segment the subcortical structures (https://github.com/lindehesse/FetalSubcortSegm_Code). The atlas was constructed using a script written in MATLAB (version R2022a), adapted from an implementation of diffeomorphic log-demons image registration (https://www.mathworks.com/matlabcentral/fileexchange/39194-diffeomorphic-log-demons-image-registration). All data analysis scripts were written in Python (3.9.6). Plots were generated using the Python seaborn package (version 0.12.1), and cortical surface maps were created using the Python-based ggseg package (version 0.1).

For manuscripts utilizing custom algorithms or software that are central to the research but not yet described in published literature, software must be made available to editors and reviewers. We strongly encourage code deposition in a community repository (e.g. GitHub). See the Nature Portfolio guidelines for submitting code & software for further information.

Data

Policy information about availability of data

All manuscripts must include a data availability statement. This statement should provide the following information, where applicable:

- Accession codes, unique identifiers, or web links for publicly available datasets
- A description of any restrictions on data availability
- For clinical datasets or third party data, please ensure that the statement adheres to our policy

This fetal brain atlas forms part of the INTERGROWTH-21st Project and will be publicly available for download (https://intergrowth21.tghn.org). Anonymised image data will be made available upon reasonable request for academic use only and within the limitations of the informed consent. Requests must be made to the corresponding author. Every request will be reviewed by the INTERGROWTH-21st Consortium Executive Committee. After approval, the researcher will need to sign a data access agreement with the INTERGROWTH-21st Consortium.

Research involving human participants, their data, or biological material

Policy information about studies with human participants or human data. See also policy information about sex, gender (identity/presentation), and sexual orientation and race, ethnicity and racism.

Reporting on sex and gender	The participating mothers are all female, and their newborn babies are described as male or female.
Reporting on race, ethnicity, or other socially relevant groupings	The study did not collect any information about ethnicity or ancestral background.
Population characteristics	The study was population-based and conducted in eight delimited urban areas: Pelotas (Brazil), Turin (Italy), Muscat (Oman), Oxford (UK), Seattle (USA), Shunyi County in Beijing (China), the central area of Nagpur (India), and the Parklands suburb of Nairobi (Kenya).
Recruitment	Participating women, who initiated antenatal care before 14 weeks' gestation, were selected based on WHO criteria for optimal health, nutrition, education and socioeconomic status needed to construct international growth standards.
Ethics oversight	The INTERGROWTH-21st Project and its ancillary studies were approved by the Oxfordshire Research Ethics Committee "C" (reference: 08/H0606/139), the research ethics committees of the individual participating institutions, as well as the corresponding regional health authorities where the project was implemented.

Note that full information on the approval of the study protocol must also be provided in the manuscript.

Field-specific reporting

Please select the one below that is the best fit for your research. If you are not sure, read the appropriate sections before making your selection.

☒ Life sciences ☐ Behavioural & social sciences ☐ Ecological, evolutionary & environmental sciences

For a reference copy of the document with all sections, see nature.com/documents/nr-reporting-summary-flat.pdf

Life sciences study design

All studies must disclose on these points even when the disclosure is negative.

Sample size	The sample size of the original fetal growth study (Lancet 2014) was based on pragmatic and statistical considerations; the latter focused on the precision and accuracy of a single extreme centile, i.e. the 3rd or 97th centile, and regression based reference limits. The availability of fetal brain volumes from that original study determined the sample to be analysed here.
Data exclusions	N/A
Replication	We validated the described patterns of fetal brain growth in 1,487 separate scans (n=1,295 separate fetuses, also born at term, and with equally satisfactory growth and neurodevelopment from early pregnancy to 2 years of age) pre-processed using the same pipeline.
Randomization	N/A
Blinding	The team analysing the fetal brain volumes were blinded to the infant neurodevelopmental outcomes.

Reporting for specific materials, systems and methods

We require information from authors about some types of materials, experimental systems and methods used in many studies. Here, indicate whether each material, system or method listed is relevant to your study. If you are not sure if a list item applies to your research, read the appropriate section before selecting a response.

Materials & experimental systems

n/a	Involved in the study
☒	☐ Antibodies
☒	☐ Eukaryotic cell lines
☒	☐ Palaeontology and archaeology
☒	☐ Animals and other organisms
☒	☐ Clinical data
☒	☐ Dual use research of concern
☒	☐ Plants

Methods

n/a	Involved in the study
☒	☐ ChIP-seq
☒	☐ Flow cytometry
☒	☐ MRI-based neuroimaging
